# Transcriptome profile of NO-induced Arabidopsis transcription factor genes suggests their putative regulatory role in multiple biological processes

**DOI:** 10.1038/s41598-017-18850-5

**Published:** 2018-01-15

**Authors:** Qari Muhammad Imran, Adil Hussain, Sang-Uk Lee, Bong-Gyu Mun, Noreen Falak, Gary J. Loake, Byung-Wook Yun

**Affiliations:** 1 Laboratory of Plant Functional Genomics, School of Applied BioSciences, Kyungpook National University, Daegu, Republic of Korea; 2 Department of Agriculture, Abdul Wali Khan University, Mardan, Pakistan; 3 Institute of Molecular Plant Sciences, University of Edinburgh, King’s Buildings, Edinburgh, UK

## Abstract

TFs are important proteins regulating plant responses during environmental stresses. These insults typically induce changes in cellular redox tone driven in part by promoting the production of reactive nitrogen species (RNS). The main source of these RNS is nitric oxide (NO), which serves as a signalling molecule, eliciting defence and resistance responses. To understand how these signalling molecules regulate key biological processes, we performed a large scale *S*-nitrosocysteine (CySNO)-mediated RNA-seq analysis. The DEGs were analysed to identify potential regulatory TFs. We found a total of 673 (up- and down-regulated) TFs representing a broad range of TF families. GO-enrichment and MapMan analysis suggests that more than 98% of TFs were mapped to the *Arabidopsis thaliana* genome and classified into pathways like hormone signalling, protein degradation, development, biotic and abiotic stress, etc. A functional analysis of three randomly selected TFs, *DDF1*, *RAP2*.6, and *AtMYB48* identified a regulatory role in plant growth and immunity. Loss-of-function mutations within *DDF1* and *RAP2*.6 showed compromised basal defence and effector triggered immunity, suggesting their positive role in two major plant defence systems. Together, these results imply an important data representing NO-responsive TFs that will help in exploring the core mechanisms involved in biological processes in plants.

## Introduction

Nitric oxide (NO), a small, gaseous, redox-active molecule, has gained attention due to its regulatory role in animal and plant physiology. Therefore, considerable research has been conducted for exploring its role in different biological processes^1,2^. Despite the attention this small molecule gained in animals for its myriad roles, the first report of NO generation in a biological system came from plants^3^ where it is reported to play a key role in physiological functions like growth, development, environmental interactions, defence^4^, stomatal regulation, and senescence^5^. Furthermore, NO’s role in mitigating abiotic stresses such as drought, cold and salt stress has also been studied^6–8,1,9^.

Despite the diverse role of NO in plant biology and the completion of various plant genome projects, the main source of NO production in higher plants is still unknown. In mammals, NO synthesis is associated with oxidative mechanisms involving NO synthase (NOS), which consists of three well-established isoforms: endothelial (eNOS), neuronal (nNOS), and inducible (iNOS)^10^. In contrast, seven possible routes have been proposed for NO production in plants^6^. The oxidative mechanisms include NO synthesis from L-arginine (L-Arg), polyamines, or hydroxyl amines while the reductive routes are dependent upon nitrite being reduced by nitrate reductase. A related protein isolated from the alga *Ostreococcus tauri* is 45% similar to human NOS enzyme. This enzyme was found to have NOS activity *in vitro* with an equally similar K_m_ for NADPH oxidation^11^. However, no such enzyme has been isolated from higher plants.

Contrary to classical signal transduction, which mostly relies on the interaction of macromolecular shapes, NO and related reactive nitrogen species (RNS) act through chemical reactions with specific targets in various proteins, resulting in covalent modifications^12^. The amino acid cysteine (Cys) of different proteins sometimes consists a low pKa sulfahydryl group that significantly enhances their susceptibility to redox-based post-translational modifications^13^. Among these redox-based post-translational modifications, NO-mediated *S*-nitrosylation is perhaps the most studied one^13^; a phenomenon in which an NO moiety is covalently attached to a solvent-exposed cysteine residue to form *S*-nitrosothiols (SNOs). Protein *S*-nitrosylation is reported to be important in cellular processes for regulating enzyme activity, protein-protein interaction, and protein localization^14^. Genes that are involved in key physiological and biochemical processes have been reported to be *S*-nitrosylated, including *NPR1*^14^, *AtSABP3*^15^, and the auxin receptor *TIR1*^16^. Recently, Hu, *et al*.^17^ showed that the proteome of *Arabidopsis GSNOR* knockout mutant *atgsnor1-3* (which reported to have significantly higher level of SNOs)^18^ contains 926 and 1195 *S*-nitrosylated proteins and peptides, respectively.

NO’s role in transcriptional control has also been reported in some studies. Following abiotic stress or pathogen ingress, cellular RNS levels increase, resulting in swift transcriptional re-programming. Microarray and RNA-seq analysis have made it easy to identify genome-wide differentially expressed genes (DEGs) in response to a particular stimulus. A number of NO-mediated transcriptomic studies have been performed so far. In a study, involving *Arabidopsis* roots and leaves, GSNO-mediated transcriptome analysis showed the differential expression of 3263 genes^19^. Similarly, microarray analysis of *Arabidopsis* roots treated with different concentrations of SNP, resulted in differential expression of 422 genes including 342 up- and 80 down-regulated genes^20^.

Transcription factors (TFs) are important regulatory proteins that control the on/off switch of genes. They contain a DNA-binding domain that binds with specific sequences in the *promoter* of different genes, thereby activating or repressing their expression to regulate various physiological functions. Therefore, higher plants have a fine-tuned, complex regulatory system consisting of transcriptional activators and repressors that manage the expression of genes involved in plant development and defence^21^. Changes in cellular NO levels can regulate the expression of important genes, including TFs such as HY5, MYB, and Trx^22^. This indicates a regulatory role for NO during physiological and molecular process via the transcriptional as well as translational control of different genes. Identification of NO-responsive TFs will help establish the regulatory role of NO in plant tolerance to abiotic stress and disease. In this study, we used high-throughput transcriptome analysis to identify 673 TFs in *Arabidopsis* leaves that showed differential expression in response to 1 mM CySNO.

In a previous study we performed RNA-Seq mediated transcriptome analysis of Arabidopsis leaves infiltrated with 1 mM CySNO generating an average of 91 million reads. CySNO infiltration induced changes in the expression of 6436 (2988 down-regulated and 3448 up-regulated) genes after 6 h^23^. This study is focused on the differentially expressed genes coding different transcription factors. Here we used both *in silico* and *in vivo* analysis to highlight the importance of TFs in plant responses to nitrosative stress.

We examined three knockout mutant lines representing three TFs *ddf1*, *rap2*.6 and *atmyb48* for their possible role in plant growth, development and defence. The DDF1 (dwarf and delayed flowering) is characterized by an AP2 domain. Overexpression of this gene results in dwarfism and delayed flowering and increase resistance to cold, drought and heat stress in Arabidopsis^24^. RAP2.6 an ERF TF family member is reportedly involved in enhanced resistance towards beet cyst nematode *Heterodera schachtii* in Arabidopsis roots through callose deposition. In addition, overexpression of this gene showed induced expression of JA-responsive genes^25^. According to TAIR annotation, AtMYB48 is involved in cell differentiation, response to salicylic acid and DNA-templated transcription factor activity. We have also used *atgsnor1-3* mutant as susceptible control due to its established role in plant immunity through SNO homeostasis in the cell^26^.

## Results

### Data mining and analysis

**Figure 1:**
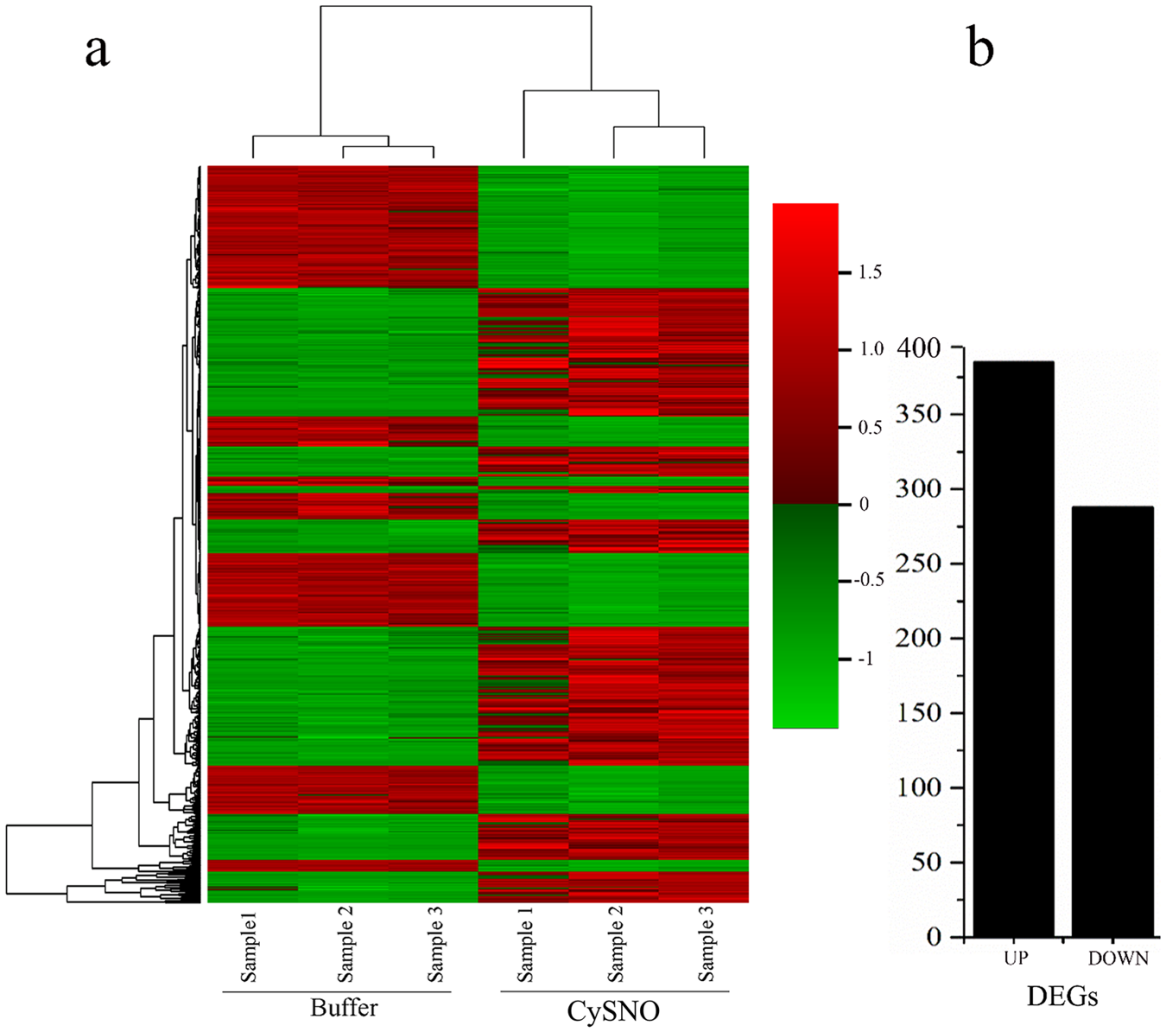
Clustering of NO-responsive TFs. (**a**) Heatmap showing the expression patterns of transcriptome-wide, differentially expressed transcription factor genes in response to CySNO. (**b**) A total of 673 TFs were identified at a Q-value < 0.05 in the transcriptome. Among these 385 were up-regulated and 288 down-regulated. The red-green colour key indicates changes in expression level (≥log2-fold change).

A total of 673 TFs that changed significantly in expression profile in response to exogenously applied 1 mM CySNO were identified by RNA-seq mediated transcriptome analysis at a cutoff Q-value of 0.05. A Heatmap generated from the expression values of controls and CySNO treated leaves (n = 3) revealed hierarchical clustering of all of the TFs genes with *P*-values < 0.05 (Fig. 1a). Of the TFs, 385 genes were up-regulated, while 288 were down-regulated in response to CySNO (Fig. 1b). An average of 23.1% TFs showed 10-fold change in their expression while 94.5% showed at least a 2-fold change, suggesting significant transcriptional changes in response to cellular NO accumulation (Supplementary Table S1). An MDS plot revealed no dispersion among control samples, however, some dispersion was found among CySNO treated samples, which may be due to the presence of TFs with very low basal expression levels (Fig. S1). A list of the top ten up- and down-regulated genes, along with their annotations, is given (Supplementary Tables S2, S3). All of the raw sequences and gene expression data were submitted to the public repository Gene Expression Omnibus (GEO) and Short Read Archive at NCBI (http://www.ncbi.nlm.nih.gov/) and are available under the accession numbers GSE81361 and SRP074890, respectively.

### GO-Enrichment analysis of NO-induced TFs

All CySNO-responsive TFs were analysed for their respective gene ontology (GO) terms and fold enrichment through the GO consortium and PANTHER classification system, using *Arabidopsis thaliana* as a reference genome. Out of 673 total TFs, 664 were successfully found in the reference *Arabidopsis* genome. GO-terms for biological processes, molecular functions and cellular components were determined. A number of TFs were involved in biological processes showing different fold enrichment (FE) (Fig. S2A; Supplementary Table S4). These included localization (3 FE), developmental processes (5.30 FE), responses to external stimuli (2.50 FE), biological regulation (9.60 FE) and biogenesis (4.30 FE); the most common categories were metabolic processes (41.90 FE) and cellular processes (30.60 FE) (Fig. S2A). Further analysis within the metabolic processes category indicated TFs involved in nitrogen metabolism (21.20 FE), biosynthetic processes (18.40 FE), catabolic processes (3.10 FE), and primary metabolic processes (54.50 FE). Detailed analysis within the cellular processes category indicated that highest FE (85.20) was observed for TFs involved in regulation of the cell cycle, followed by cell communication (11.10 FE) (Fig. S2A). Similarly, TFs associated with binding activity (75.40 FE) and catalytic activity (19.60 FE) were the dominant categories in terms of FE in GO terms for molecular functions. Further analysis within the catalytic activity category revealed key catalytic activities including transferase (46.30 FE), hydrolase (31.70 FE), ligase (7.30 FE), and enzyme regulation (7.30 FE) (Fig. S2B). Within the binding activity category, nucleic acid binding (68.80 FE), protein binding (17.60 FE), and chromatin binding (13.20 FE) were the major categories (Fig. S2B). GO analysis for cellular components revealed TF genes localized to membranes (5.20 FE), macromolecular complexes (13.90 FE), organelles (35.80 FE), and other cellular parts (45.10 FE). The highest FE for TFs related to organelles were observed for those localized in the nucleus (93.80 FE, Fig. S2C).

### NO responsive major transcription factor families

**Figure 2:**
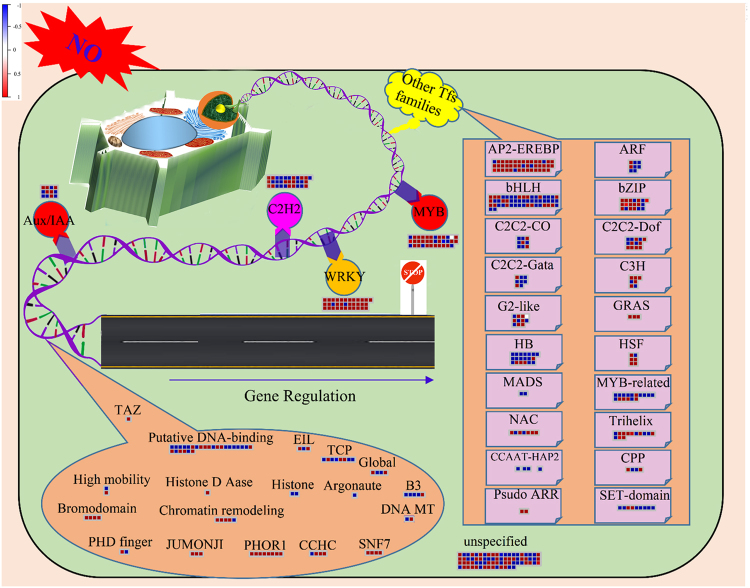
MapMan analysis of NO-responsive TFs involved in different pathways. All the transcription factors that showed differential expression to CySNO were analysed for functional classification into different pathways using TAIR 10 (Aug 2012) as a reference genome database in MapMan. This figure is a schematic representation of gene regulation through different transcription factors and major transcription factor families that showed differential expression in response to CySNO. The red-blue key indicates up- and down-regulated genes, respectively. The illustrations were made using Microsoft PowerPoint (2013) and CorelDRAW X7.

All of the CySNO-responsive TFs were analysed to identify major TFs families using MapMan. A number of well-known, key transcription factor families were induced in response to NO accumulation. The basic helix-loop-helix (bHLH) was the dominant TF family, having 53 TFs (Fig. 2). bHLH proteins are a superfamily of TFs that bind to DNA as a dimer and are characterized by the presence of a ~ 60 amino acid bHLH domain. This domain is well-characterized in mammals^27^; in plants, however, it is not well understood. AP2 (APETALA2) and EREBPs (ethylene-responsive element binding protein) was the second major TF family, with 44 TFs, that showed differential expression to CySNO (Fig. 2). AP2- EREBP are the archetypic members of this family, found only in plants^28^. It is a large, multi-gene family and is the key regulator of many developmental processes and responses to various environmental and biotic stimuli^28^.

We also identified 36 MYB and 16 MYB-related TFs in the CySNO-responsive transcriptome analysis (Fig. 2). MYB family genes have been reported in a number of plant and animal species including Arabidopsis, maize, populous, soybean and brassica for their diverse roles reviewed in^29^. MYB has been implicated for its role in ABA signalling and interaction with other TFs in plants^29^. The WRKY TF family is one of the largest TF families found in plants. Members of this family are characterized by presence of at least one conserved WRKY domain. We found that out of 74 total WRKY TFs found in Arabidopsis, 33 of these showed differential expression in response to CySNO (Fig. 2). The C2H2 Zinc-Finger TF family is another TF family found in plants that regulates genes involved in growth and development, as well as both biotic and abiotic stress conditions^30^. Of 211 C2H2 TF genes in Arabidopsis, 31 showed differential expression in response to CySNO (Fig. 2). Auxin/indole-3-acetic acid (Aux/IAA) proteins are short-lived TFs that are responsible for auxin-dependent transcriptional regulation. We found 12 Aux/IAA family members that showed differentially expression in response to NO-donor (Fig. 2).

Similarly, other transcription factor families showed differential expression in response to CySNO (Fig. 2) including: bZIP (basic region/leucine zipper motif), which regulates light and defence signalling responses in plants^31^; ARF (Auxin responsive factor family); C2C2-Gata, reportedly involved in regulation of light or circadian rhythms^32^; C3H; HB (home box), a 60 amino acid peptide initially discovered in *Drosophila* that regulates embryogenesis and pathogenesis^33^; the SET-domain, recognized as having methyl-transferase activity^34^; the NAC domain, which is involved in transcriptional re-programming during plant biotic and abiotic stress responses^35^; MADS box, involved in plant developmental processes^36^; Trihelix, involved in the regulation of light, pathogen attack, and salt responses in plants^37^; GRAS, a key player of GA signalling, thus regulating growth and development^38^; and PHOR1, a U-box component of GA signalling with a role in proteasome degradation^39^. However, a large number of TF genes (74) were mapped as unspecified (Fig. 2).

### Transcription factor genes putatively involved in cellular processes and defence

**Figure 3:**
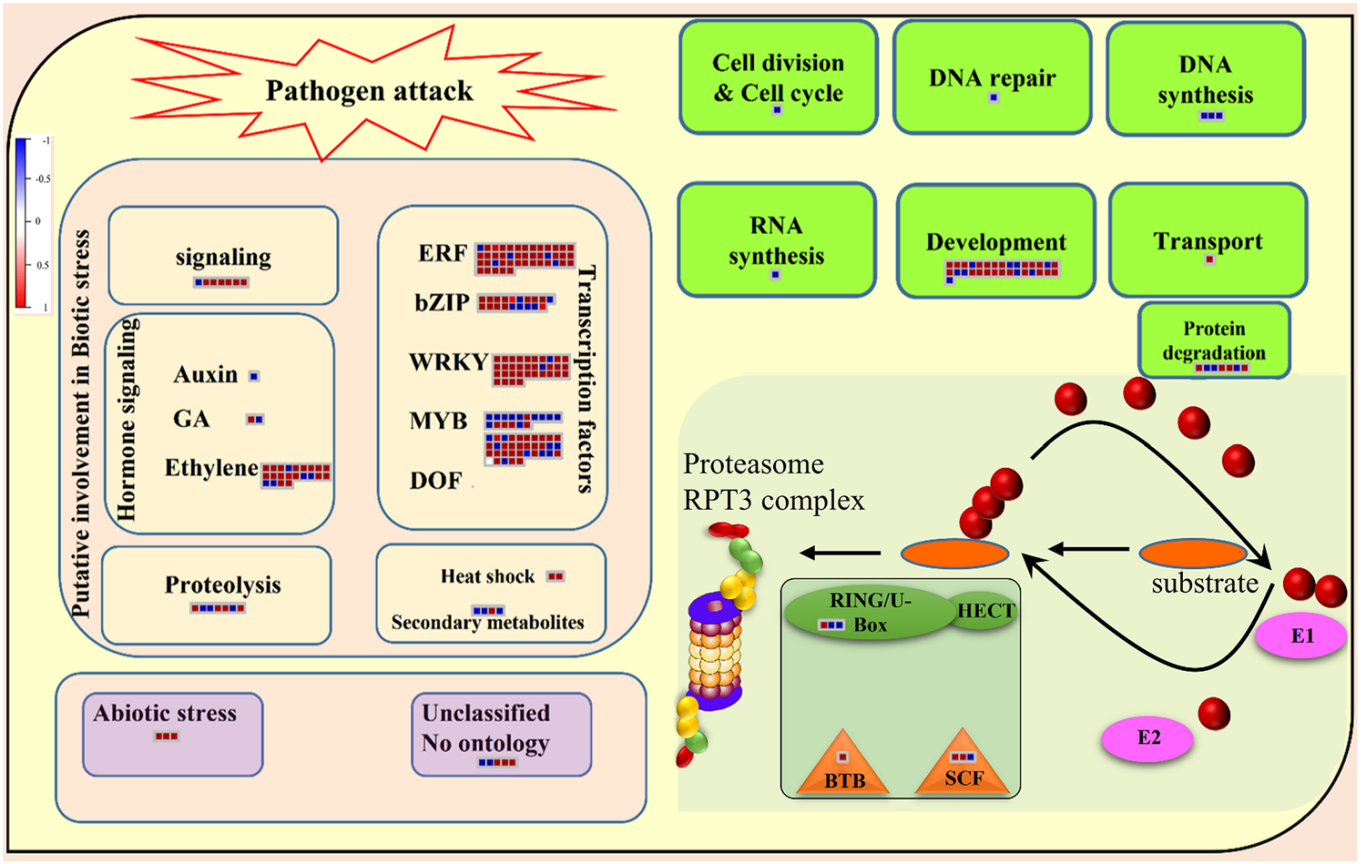
An average of 30% TFs were involved in defence. Functional classification of TFs involved in different cellular process and defence. Out of all NO-induced TFs, 193 were involved in defence and 31 in development. The red-blue colour key indicates up- and down-regulated genes, respectively. The bottom-right section of the figure represents ubiquitin proteasome system (UPS) that acts in ATP-dependent manner with Regulatory Particle Triple-A ATPase 3 (RPT3). Proteins are targeted to the proteasome by tagging with small ubiquitin protein (red balls) through a 3 step processes. First E1 ubiquitin-activating enzyme is covalently linked with an ubiquitin protein which is then transferred to a E2 ubiquitin-conjugating enzyme. This activates the E3 ubiquitin ligase complex having RING U-box and HECT (homologous to E6-assoicated protein C terminus) domain proteins that mediates transfer of ubiquitin to lysine residue in the substrate thus marking them for proteasomal degradation. About 7 different NO-responsive TFs were mapped to the E3 ubiquitin-ligase complex suggesting NO role in UPS.

Almost 30% (201/673) of the CySNO-responsive TFs showed putative involvement in biotic and abiotic stress responses (Fig. 3). Out of these, 95.5% (193/201) were related to biotic stress. A heatmap showing the expression of TFs involved in defence is also given (Supplementary Fig. S3). These include a variety of TFs like ERF, bZIP, WRKY, MYB and DOF (the detail of these TFs is given in the “major transcription factors” section). Among these, twenty-five TFs were mapped to biotic-stress-mediated hormone signalling, seven were mapped to proteolysis, and four were putatively involved in biotic-stress-mediated secondary metabolite production (Fig. 3). Similarly, among other cellular processes, thirty-one TFs were putatively involved in development, seven in protein degradation, three in DNA synthesis, and a single TF each in cell division and cell cycle, DNA repair, RNA synthesis, and transport (Fig. 3).

### TFs putatively involved in biotic stress signalling

We identified seven TFs that were putatively involved in signalling during stress conditions using MapMan (Fig. 3). Among these, 6 were up-regulated while only one was down-regulated in response to CySNO. These include AT1G35160 (GF14PHI), involved in brassinosteroid signalling; At2g32250 (FRS2), involved in light signalling; At3g59220 (PRN); At4g09570 (CPK4), involved in ABA signalling; and At5g60890 (AtMYB34), involved in the tryptophan pathway, according to TAIR (https://www.arabidopsis.org/) annotations. The single down-regulated TF involved in signalling showed a more than 9-fold change in expression level and is negative regulator of the GA signalling pathway (Fig. 3).

### TFs putatively involved in growth and development

The role of TFs in regulating plant growth and development has already been studied in detail^40^. We identified thirty-one (17 up- and 14 down-regulated) TFs involved in growth and development that showed differential expression to CySNO (Fig. 3). These include members of the GRAS, NAC, AP2/EREP, and MADS TF families. The role of these TF families in growth and development has been already reported^36^. The highest-fold change was in At1g52890 (NAC TF), which showed a more than 150-fold change in expression after CySNO. The expression of this gene is induced by drought, high salinity, and ABA^41^. We also found a SQUAMOSA promoter-binding protein-like (SPL) TF (At1g76580) that is involved in key biological processes like plant phase transition, flower and fruit development, GA signalling, sporogenesis, plant architecture, and response to copper and fungal-released toxins^42^. Among down-regulated TFs was Origin Recognition Complex subunit 2 (AtORC2), which is involved in initiation of DNA replication during the cell cycle.

### TFs related to protein degradation

Swift degradation of particular target proteins by proteasome-dependent pathways is an important part of many cellular regulatory processes/mechanisms. We identified seven (4 up- and 3 down-regulated) TFs putatively involved in protein degradation (Fig. 3). This indicates a regulatory role of NO in protein degradation. Among the up-regulated TFs in *Arabidopsis*, MATH-BTB domain proteins (*AtBPM1*) have a direct interaction with targets of proteasomal degradation. Another TF, *ADO2* (At2g18915), whose expression was changed by two fold, encodes a member of the F-box protein family (including 2 proteins in *Arabidopsis*, ZTL and FKF1) that regulates circadian rhythms (Supplementary Table S1). Similarly, among down-regulated TFs, we identified a jumonji TF that has a putative function in Zn binding (Supplementary Table S1). These TFs are thus good candidates for NO-regulated metal biology.

### *RAP2*.6 negatively regulates shoot length while *DDF1* negatively regulates root length under nitrosative stress

**Figure 4:**
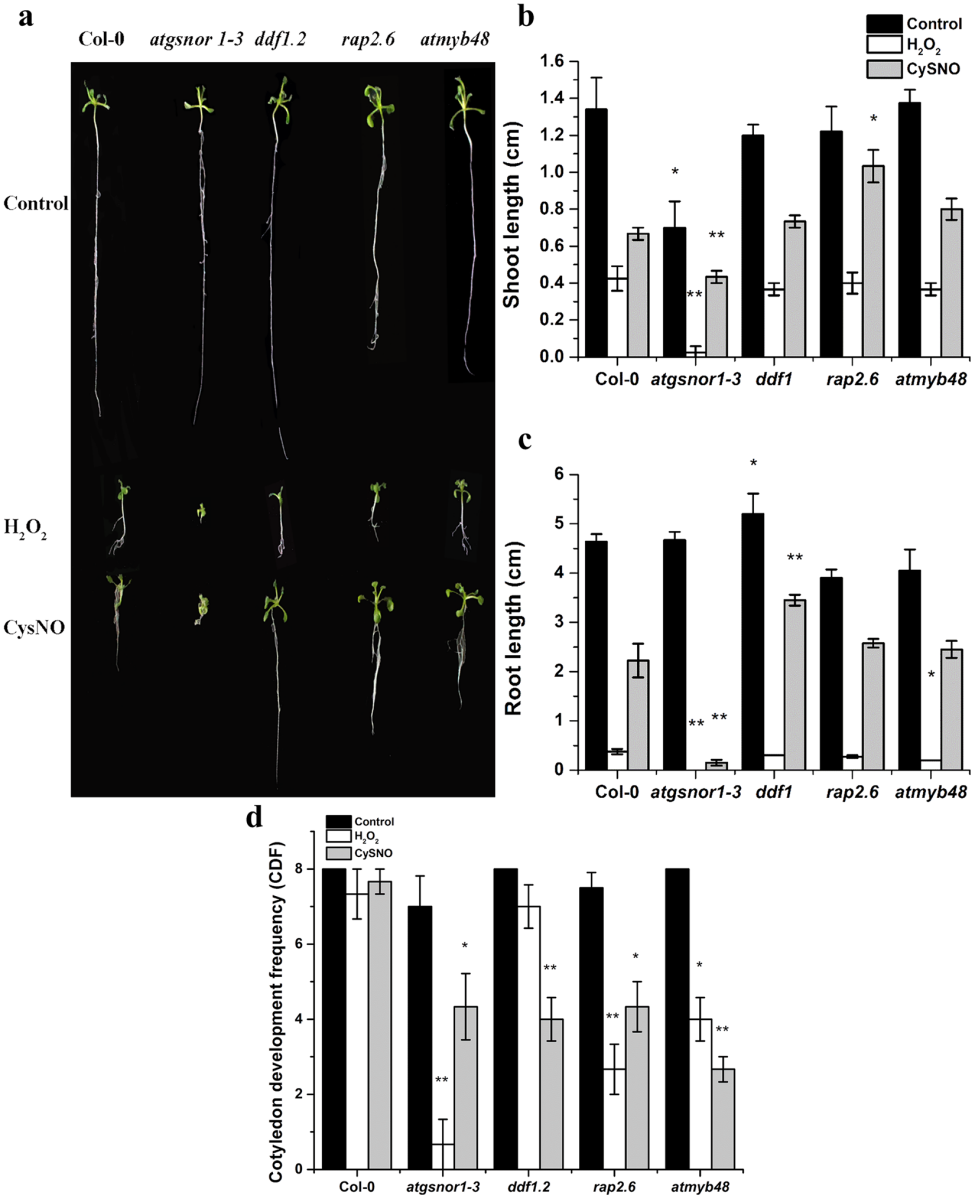
*ddf1*, *rap2*.*6 and atmyb48* response to oxidative and nitrosative stress. (**a**) Phenotypes of the indicated genotypes under control, oxidative (H_2_O_2_) and nitrosative (CySNO) stress conditions. (**b**) Shoot length and (**c**) root length and (**d**) cotyledon development frequency (CDF) compared to WT control. All the data points represent the mean of three replicates with 8 plants per replicate. The experiment was repeated three times with similar results. Error bars represents standard error (±SE). Significant differences are shown by asterisks (Student’s *t*-test with 95 and 99% confidence levels).

To identify a possible role of NO-induced TFs in growth and development, the selected knockout mutant lines *ddf1*, *rap2*.6, and *atmyb48* were screened for their growth performance under oxidative and nitrosative stress induced by 2 mM H_2_O_2_ and 1 mM CySNO respectively. Under control conditions, there was no significant difference in cotyledon development frequency (CDF) among all mutant lines compared to wild type (WT) (Fig. 4d); however, under nitrosative stress conditions, all mutant lines showed a significant reduction in CDF compared to WT (Fig. 4d). Similarly, under oxidative stress condition, all mutant lines except *ddf1* showed a reduction in CDF (Fig. 4d). Furthermore, under control and oxidative stress conditions, shoot length measurements revealed no significant difference between mutant lines and WT, except *atgsnor1-3* (Fig. 4a,b). The *ddf1* line showed significantly higher root length compared to WT under control and nitrosative stress conditions (Fig. 4a,c); however, under oxidative stress conditions, only *atmyb48* showed a significant reduction in root length compared to wild type (Fig. 4c).

### Nitric oxide responsive TFs regulate ROS-RNS cross-talk

**Figure 5:**
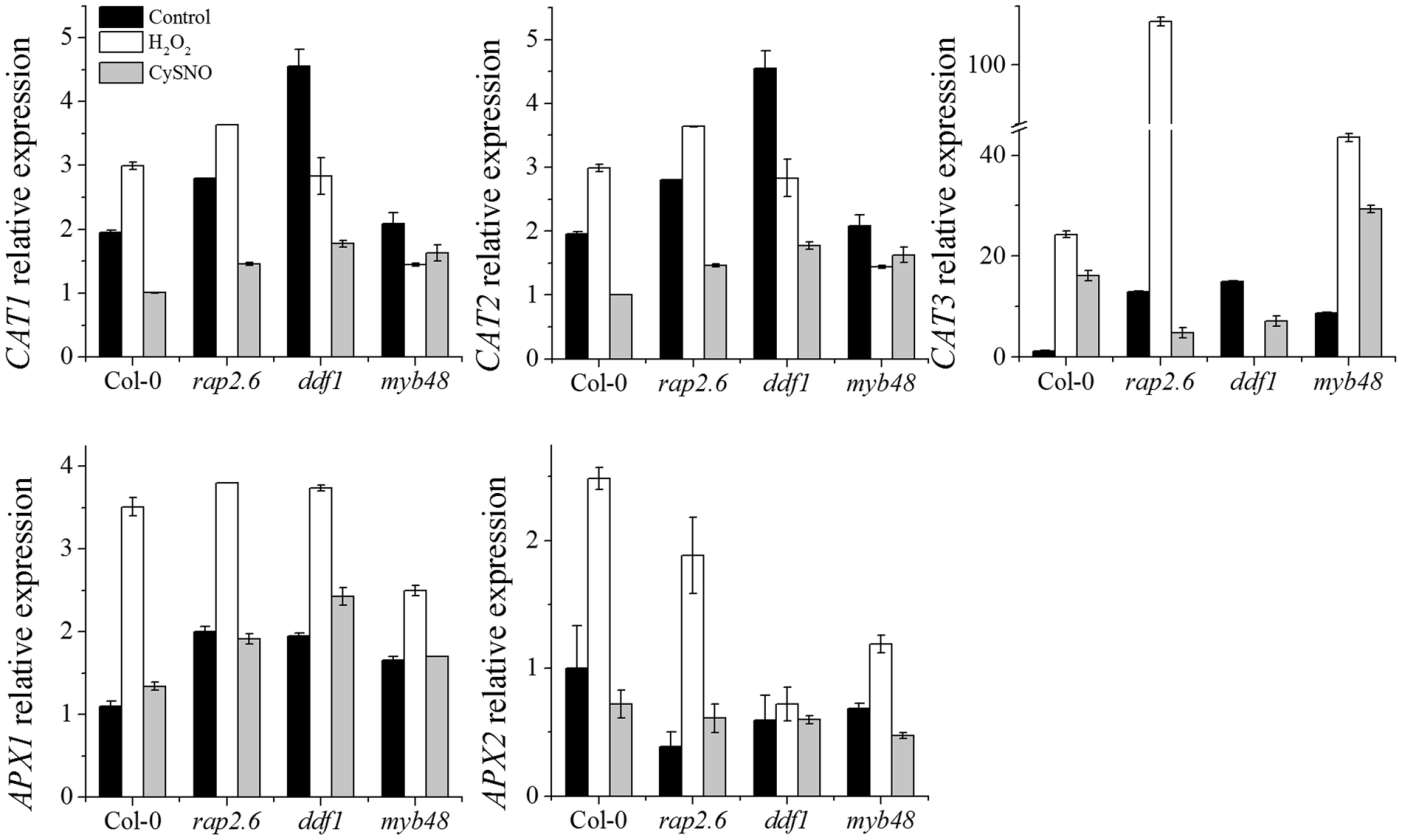
Transcript accumulation of key marker genes involved in oxidative stress. The expression of selected genes involved in regulation of oxidative stress regulation were analyzed through quantitative real-time PCR. Each data point is the mean of three replicates and error bars represent standard error (±SE).

**Figure 6:**
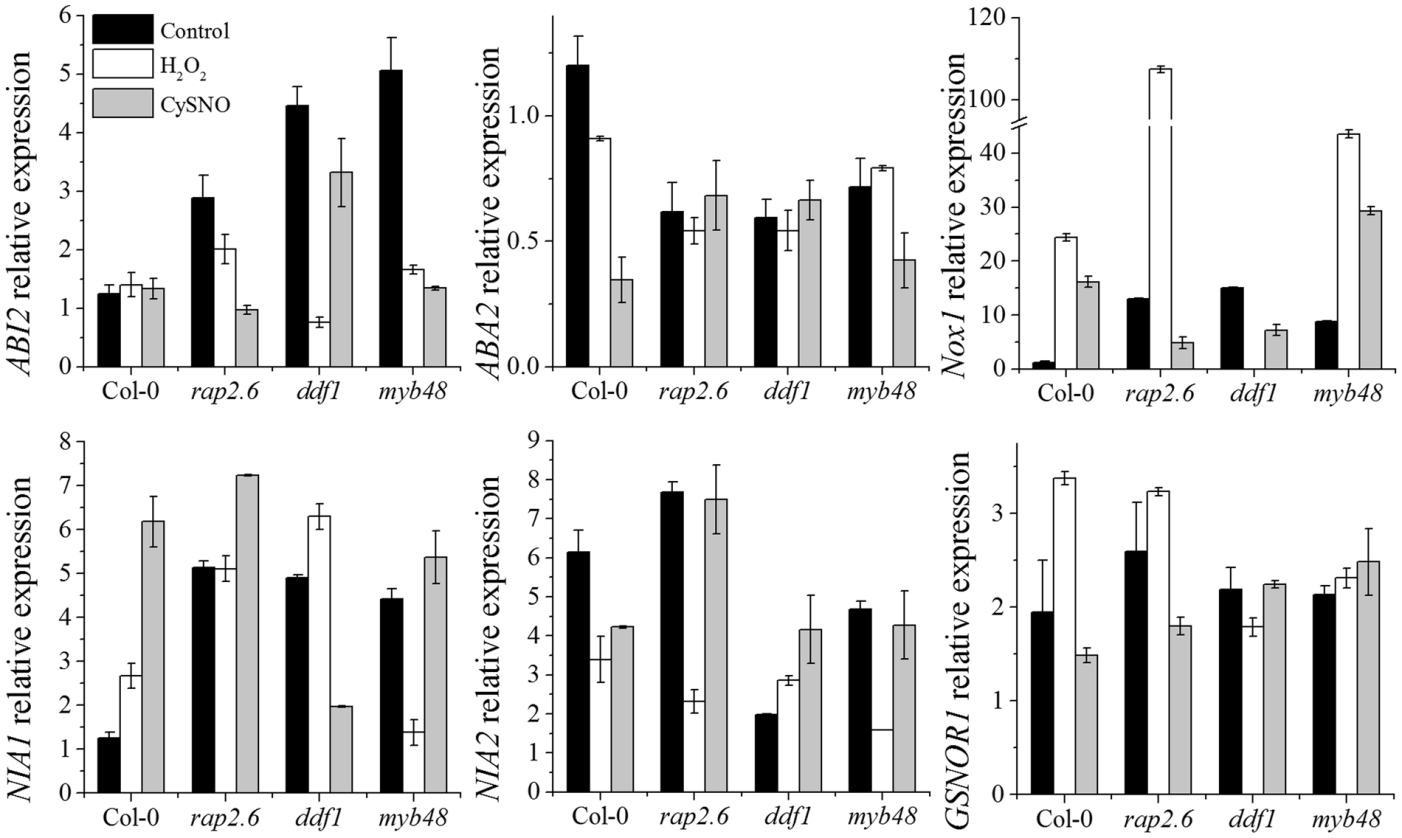
Relative expression of key marker genes regulating nitrosative metabolism. Expression of selected genes involved in the regulation of nitrosative cascade were analyzed through quantitative real-time PCR. Each data point is the mean of three replicates and error bars represent standard error (±SE).

Bulk of literature reports extensive crosstalk between reactive oxygen and reactive nitrogen species in living cells; the ultimate aim of which is to maintain a critical balance between these two redox-pools for smooth functioning. The transcription factor knock out mutant plants *rap2*.6, *ddf1* and *myb48* showed an interesting response when grown on oxidative and nitrosative stress media (Fig. 4). When grown on basal medium all the mutant plants showed significantly higher expression of the *NADPH oxidase 1* (*NOX1*) as compared to Col-0 indicating that these mutants would accumulate more ROS. However, as expected the mutant plants also showed significantly higher expression levels of genes encoding the three catalase enzymes (*CAT1*, *CAT2* and *CAT3*) and the gene encoding the superoxide dismutase enzyme (*APX1*) on basal media (Fig. 5). The mutant plants also showed a significantly higher expression of *NIA1* the enzyme responsible for the production of nitric oxide *via* the reductive pathway (Fig. 6). This indicates an increase in the production of RNS in order to counterbalance the increasing cellular ROS.

Furthermore, *NOX1* expression in *rap2*.*6*, and *myb48* dramatically increased on MS medium containing H_2_O_2_ whereas, it diminished in the *ddf1* mutant (Fig. 6). However, on CySNO medium the expression of this gene decreased significantly in *rap2*.6 and *ddf1* whereas, increased in *myb48*. Similarly, the expression of *NIA1* increased significantly in all the mutant plants grown on CySNO medium as compared to those grown on H_2_O_2_ and basal medium whereas, *NIA2* expression showed a varied response indicating redundancy in the response of these two isoforms of the nitrate reductase enzyme (Fig. 6). Expression of *GSNOR1* (which controls global levels of S-Nitrosothiols in plants) was significantly higher in mutant plants grown on H_2_O_2_ medium but lower in mutant plants grown on CySNO medium (Fig. 6). Similarly, significantly higher expression levels of *APX1* were observed in plants grown on H_2_O_2_ medium but lower in plants grown on CySNO medium whereas, *APX2* showed a varied response (Fig. 5).

Reactive oxygen and nitrogen species have been known to regulated drought tolerance in plants by regulating ABA biosynthetic and signaling pathways and *via* ABA mediated stomatal closure. Nitrate reductase-mediated NO generation is required for ABA-induced stomatal closure^43^. The *rap2*.*6*, *ddf1* and *myb48* plants showed significantly higher basal expression level of *ABI2* as compared to WT plants but the expressions of *ABA2* were significantly lower than WT plants. However, *rap2*.*6* and *myb48* showed higher expression of *ABI2* when grown on media containing H_2_O_2_ but lower expression on media containing CySNO. Inversely, *ddf1* showed lower expression of *ABI2* on H_2_O_2_ medium but higher expression on CySNO medium (Fig. 6). These results indicate that NO-responsive TFs play an important role in regulating the cross talk between reactive oxygen and nitrogen species.

### *RAP2*.6 and *DDF1* positively regulates basal disease resistance

**Figure 7:**
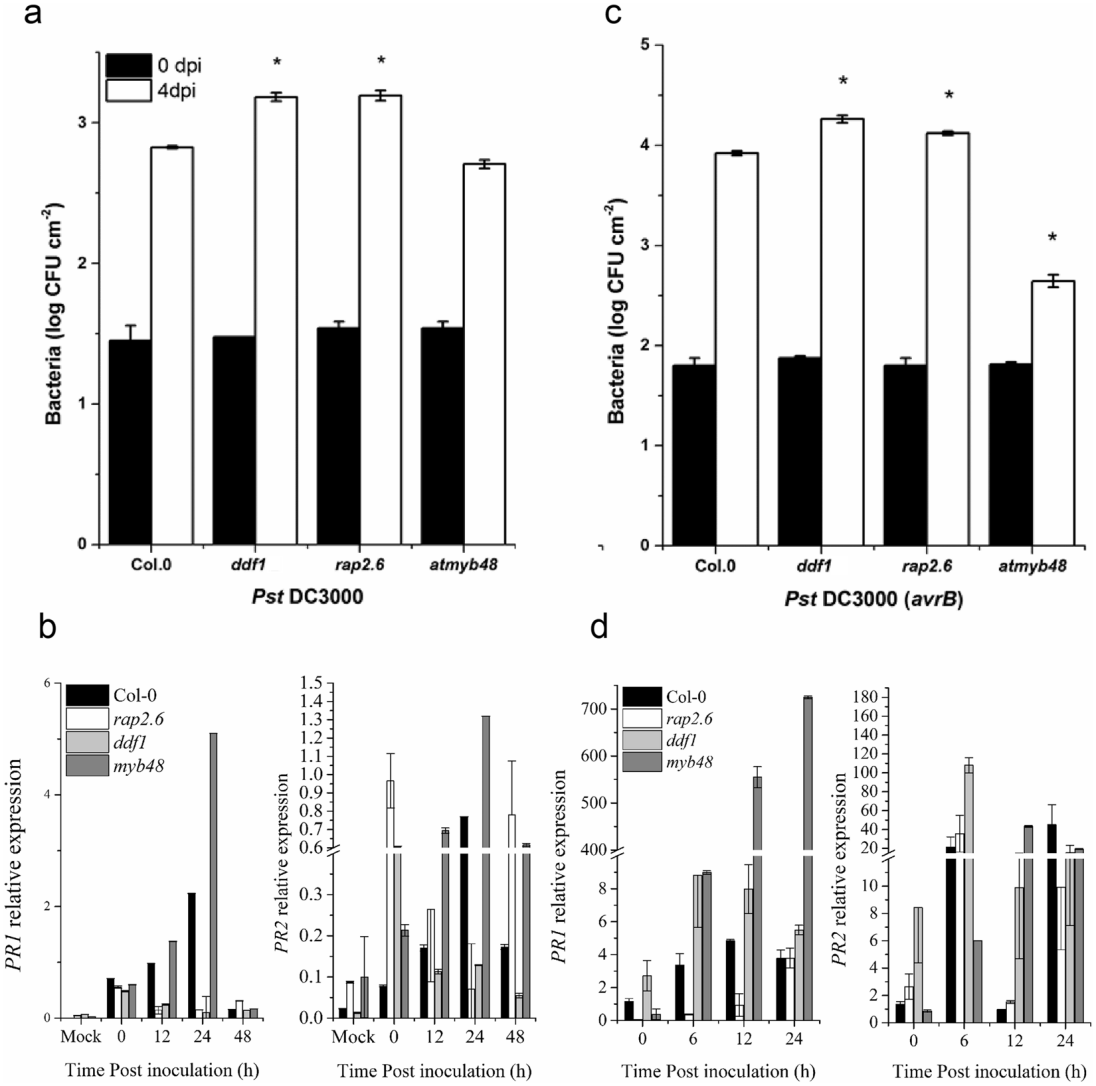
AtMYB48 negatively regulates basal defence and effector triggered immunity (ETI) while DDF1 and RAP2.6 positively regulate them. Pathogen growth from leaves pressure infiltrated with (**a**) virulent *Pst* DC3000 (**b**) PR gene expression in response to virulent *Pst* DC3000 inoculation. (**c**) Pathogen growth in response to *Pst* DC3000 (*avrB*) at 5 × 10^5^ colony forming units (CFU) mL^−1^ (unless stated otherwise) after 0 and 4 days of inoculation (d) PR gene expression after attempted *Pst* DC3000 (*avrB*) inoculation. Error bars represent ± SE (n = 3). Significant differences are represented by asterisks (student *t*-test with 95% confidence interval).

The basal defence system uses pathogen-associated molecular patterns (PAMPs) that serve to limit the growth of virulent pathogens within the susceptible host. To examine if AtMYB48, RAP2.6, and DDF1 are required for basal defence, we inoculated plants with *Pst* DC3000, which is virulent on the *Arabidopsis* Col-0 accession. Both *rap2*.*6* and *ddf1* showed increased susceptibility as shown by significantly higher pathogen growth compared to WT, while *atmyb48* showed resistance towards the virulent *Pst* DC3000 (Fig. 7a). Thus, mutations in *RAP2*.6 and *DDF1* compromise basal defence response while those in *AtMYB48* induce it. These results were further supported by real time PCR analysis of *PR1* and *PR2* genes. *rap2*.6 and *ddf1* plants showed significantly lower expression of these genes after 12 and 24 hours of inoculation with *Pst* DC3000 (Fig. 7b). Whereas, *myb48* showed significantly higher expression of *PR1* and *PR2* following *Pst* DC3000 inoculation (Fig. 7b) indicating that MYB48 negatively regulates basal defence system in Arabidopsis.

### AtMYB48 negatively regulates effector-triggered immunity

We examined the impact of *AtMYB48*, *RAP2*.6 and *DDF1* mutations during effector-triggered immunity (ETI; *R* gene-mediated resistance). The major Arabidopsis *R* genes encode NBS-LRR (nucleotide binding sites -lucine rich repeats) proteins^44^. NB-LRR proteins have the capability to recognize pathogens from diverse kingdoms and activate defence responses called ETI. We determined if AtMYB48, RAP2.6 and DDF1 are required for ETI by examining the growth of pathogenic *Pst* DC3000 expressing *avrB* within the inoculated leaves. WT plants demonstrated ETI against *Pst* DC3000 (*avrB*) due to the presence of the CC-NBS-LRR protein RPM1^45^. The mutant lines *rap2*.*6* and *ddf1* showed increased pathogen growth compared to WT (Fig. 7c) with a concomitant lower expression of *PR1* and *PR2* genes (Fig. 7d). Conversely, *atmyb48* exhibited resistance to *Pst* DC3000 (*avrB*) compared to WT with significantly higher expression levels of *PR1* and *PR2* genes within the first 12 hours of inoculation (Fig. 7d). This indicates that *AtMYB48* negatively regulates ETI, while *RAP2*.6 and *DDF1* positively regulate it.

### qRT-PCR validation

**Figure 8:**
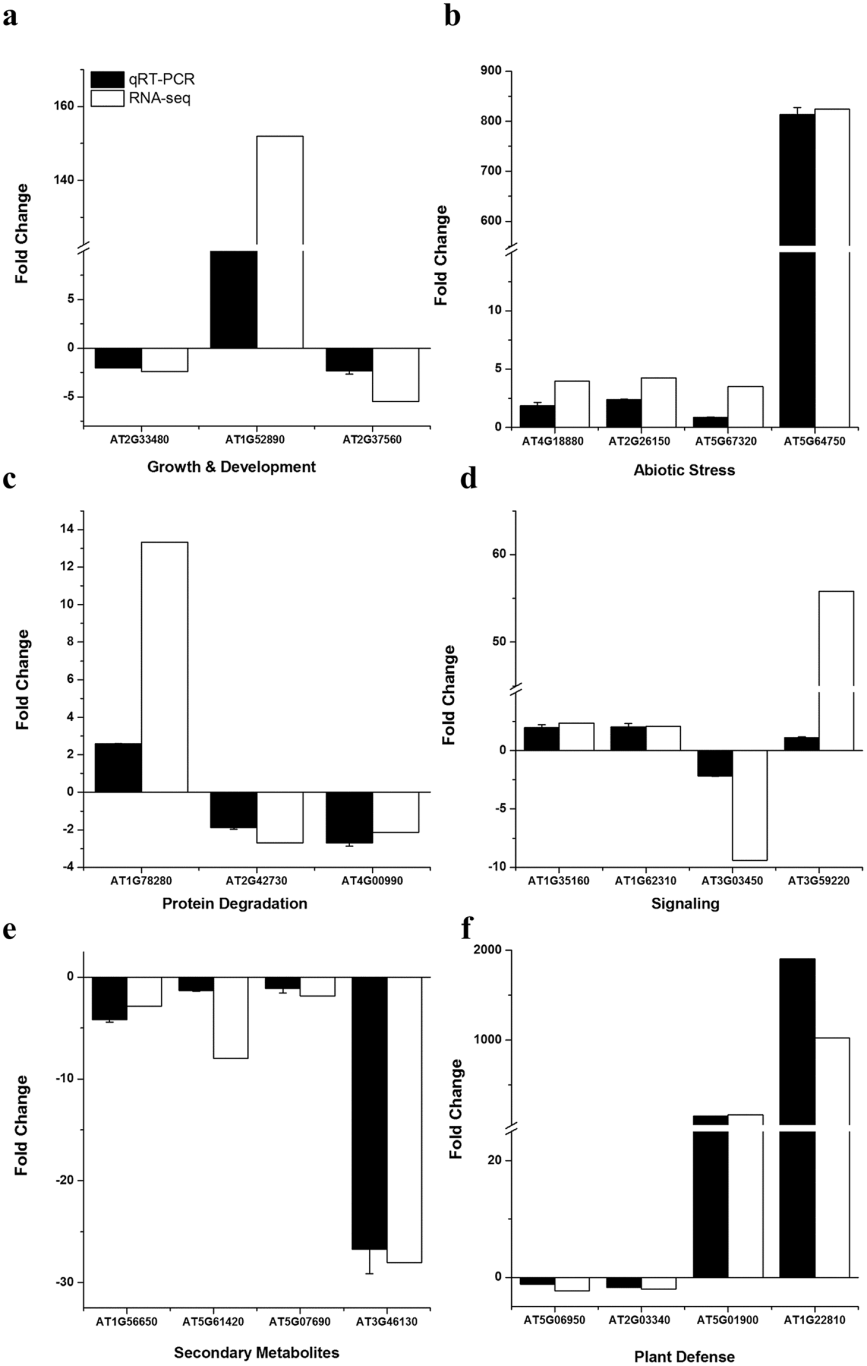
Validation of RNA-seq data by qRT-PCR analysis. A total of 22 genes involved in different biological processes that showed differential response to CySNO in RNA-seq analysis were selected for confirmation through qRT-PCR. These genes include those involved in (**a**) growth and development, (**b**) abiotic stress, (**c**) protein degradation, (**d**) signalling, (**e**) secondary metabolite production, and (f) plant defence. The correlation coefficient (R) of 0.951 indicates that qRT-PCR results are significantly correlated with RNA-seq analysis. Each data point is the mean of three replicates and error bars represents standard error (±SE).

To further validate the CySNO-mediated transcriptional changes in TF genes, we analysed the transcript accumulation of selected representative genes after 6 h of 1 mM CySNO infiltration in *Arabidopsis* leaves, using qRT-PCR. These genes were involved in different key biological process such as growth and development, abiotic stress, protein degradation, signalling, secondary metabolite production, and plant defence (Fig. 8a–f and Supplementary Fig. S5). Fold change was calculated from qRT-PCR data and correlated with RNA-seq results using Microsoft Excel (version 2016; https://www.office.com/). Our correlation coefficient of 0.951 indicates a high, significant correlation between the RNA-seq and qRT-PCR analyses (Fig. 8a–f).

## Discussion

Nitric oxide (NO) is a major signalling molecule that regulates a plethora of biological process in both plants and animals. After its initial identification in mammalian systems^1^, scientists started to hunt for its presence in plants. Since its first report in a plant system, extensive research has been conducted on different aspects of NO biology. Changes in NO-mediated cellular redox status can affect the expression of important genes like peroxidases and catalases, receptor proteins^16^, protein kinases, and transcription factors like MYB, Trx and HY5^22^. CySNO is a well-known and widely used NO source due to its low molecular weight and high diffusivity into plant tissues compared to other NO donors. Reports suggest that many NO-modulated genes are regulatory proteins or transcription factors (TFs)^22^. Therefore, studying global changes in the expression of TFs will help clarify the underlying mechanisms involved in the regulation of key biological processes.

A large-scale CySNO-mediated RNA-seq analysis, covering almost 176 million reads, was performed to describe transcriptional changes in response to nitrosative stress. The details of the differentially expressed genes (DEGs) and their analysis has already been reported^46^. A total of 98.6% (664) of the TFs were successfully mapped to the *Arabidopsis* genome. The remaining seven TFs may be a potential source of novel TFs in *Arabidopsis*.

GO analysis indicated TFs putatively involved in biological process regulation such as cell communication, cell cycle regulation, developmental processes, biogenesis, biosynthetic processes, and nitrogen metabolism (Fig. 2a). These results are consistent with a number of published reports claiming the involvement of NO in regulating whole plant growth, primary root development, hypocotyls, shoot, germination and leaf senescence [reviewed in^6^]. Similarly, GO annotation for Molecular Function indicated TFs putatively involved in two major categories, binding activity (75.40 FE) and catalytic activity (19.60 FE) (Fig. S2B). The large number of TFs classified to the binding category is because of their ability to bind with DNA at specific sequences in the promoter region^22^. Crosstalk between NO and transcription factor binding sites (TFBSs) has also been studied, and eight families of TFBSs, including WRKY and ocs element-like sequences, were found at least 15% more often in the promoter regions of NO-responsive genes compared to 28447 control genes in *Arabidopsis*^22^. TFs classified to catalytic activity confirm the putative involvement of NO-responsive TFs in the regulation of enzyme activity. Changes in the expression level of important enzymes in response to CySNO were also evident in our previous study involving RNA-seq and qRT-PCR analyses. These included disulfide isomerases, mono- and di-hydro ascorbate reductase, glutathione peroxidases, oxido-reductases, catalases (CAT2 and CAT3), carboxy methyltransferase, MAP kinases, MAPK phosphatases etc^46^.

In this study, we found many important NO-induced transcription factor families, such as bHLH, AP2-EREBP, MYB, WRKY, C2H2, Aux/IAA, bZIP, and DOF (Fig. 2). All these TFs regulate key biological processes like light signalling; abiotic and biotic stress responses; ABA, Auxin, and GA signalling; salt response; pathogenesis; etc. Some of these TFs are already known for their regulatory role under abiotic and/or biotic stress, such as NAC^35^ MYB, WRKY, C2H2 Zinc-finger reviewed in^29^ etc. A promoter analysis of NO-responsive genes showed many TF binding sites^22^. These include WRKY, GBOX, DOFF, HEAT, MADS, MYB, and CAAT, indicating extensive crosstalk between NO and these TFs. The remaining 74 unspecified TFs may be a good source for uncovering the function of these uncharacterised genes.

The production of NO in response to biotic stress like pathogenesis is well studied. This results in changes in cellular redox tone, which is highly toxic for plants. Plants have a finely tuned system to convert highly reactive and toxic NO into a mobile, non-toxic, and easily available form GSNO. There is an intricate balance between NO production and its conversion to GSNO, catalysed by a key enzyme, GSNOR. Mutations in this gene result in compromised basal defence and *R*-gene mediated resistance^26^. Our results showed almost 30% of total NO-responsive TFs were related to stress tolerance, among which 95.5% were related to biotic stress (Fig. 3). These include members of the ERF, bZIP, WRKY, MYB and DOF families, among others (Fig. 4, Supplementary Fig. S3). As an example, the bZIP TF is reportedly involved in the regulation of abiotic and biotic stress^47^. A class of bZIP-binding elements associated with defence is formed by octopoine synthase (OCS). These OCS-like elements are imperative for the expression of specific pathogenesis-related genes such as *PR1* in *Arabidopsis*^48^. This was further supported by the finding that the *Arabidopsis* TGA/ocs element-binding factor interacts with NPR1, the main player of the SA-mediated defence signalling pathway^49^.

Experiments were also conducted to confirm the regulatory role of TFs in growth, development, and plant defence. Our results suggested a significant increase in root length for mutant line *ddf1* under control and nitrosative stress conditions compared to WT. Literature reports reveal that overexpression of *DDF1* results in a dwarf phenotype^24^. This suggests that DDF1 negatively regulates plant growth, which is also evident from our results (Fig. 4a,b). Furthermore, *atmyb48* had a significant reduction in root length under H_2_O_2_-mediated oxidative stress compared to wild type, suggesting a positive regulatory role of AtMYB48 in root development (Fig. 4a,c). This might be because most MYB TFs are involved in abiotic stress tolerance, particularly to oxidative stresses such as drought, salinity, and osmotic stress. Reports suggest that MYB TFs such as *AtMYB2*, *AtMYB74*, and *AtMYB102* are up-regulated in response to drought and various other abiotic stresses^50^.

We also investigated role of these TFs in basal defence and ETI by pathogen inoculation with *Pst* DC3000 (virulent) and *Pst* DC3000 (*avrB*) respectively. The mutant lines *ddf1* and *rap2*.*6* showed compromised basal defence and ETI and showed significantly higher pathogen growth compared to WT, indicating a positive regulatory role of these genes in basal and ETI (Fig. 7a,c). These results are in accordance with previous results that showed a regulatory role of *rap2*.*6* in response to type III secretion system of virulent and avirulent strains of *P*. *syringae* and to nematode pathogens^25^. Reports suggested that *RAP2*.*6* has been involved in responses to various biotic and abiotic stress conditions, suggesting its role in the regulation of these stresses^51^.

We also validated our RNA-seq results by selecting representative TF genes involved in key biological processes through qRT-PCR (Fig. 8). The high coefficient value (0.95) indicates high fidelity to the RNA-seq results^46^. NO-induced TFs may bind to enhancer proteins, enabling the recruitment of RNA polymerase for transcription initiation (Supplementary Fig. S5). Alternatively, TFs may also bind to repressor proteins, blocking RNA polymerase attachment and hence inhibiting transcription. Thus, depending upon the type, TFs may activate or repress transcription^52^. We identified genes involved in various biological processes with an RNA-seq mediated transcriptome of *Arabidopsis* leaf in response to 1 mM CySNO and validated them through qRT-PCR (Fig. 8, Supplementary Fig. S5), suggesting that TFs plays key role in the mechanistic control of diverse cellular processes.

Collectively, this information regarding NO-induced transcription factors will help in portraying a clearer picture of NO-mediated transcriptional control of different regulatory genes. The biological data have confirmed significant crosstalk between oxidative and nitrosative stress conditions, in addition to plant defence.

## Methods

### Plant material, growth and treatment

Seeds of Wild Type (WT) *Arabidopsis thaliana* (accession Col-0) were obtained from the Nottingham *Arabidopsis* Stock Centre (NASC) (http://arabidopsis.info/) and grown under long day conditions (16 h of light and 8 h of dark) at 23 ± 2 °C. The plants were infiltrated with 1 mM CySNO on the abaxial side of leaves at rosette leaf stage (4-week-old plants, unless stated otherwise). The 1 mM CySNO concentration is a standard dose for studying the effect of nitrosative stress on the phenotype and the expression of a plethora of genes, of *Arabidopsis thaliana*. The same dose have been used in different published reports e.g.^53,54^. The control plants were infiltrated with buffer. Leaf samples were collected after 6 h of infiltration (after screening for optimum effect over time; supplementary Fig. S4).

### RNA and cDNA library construction

An RNeasy® Plant Mini Kit (Qiagen) was used to extract RNA from leaf samples (3 replicates) using the standard protocol suggested by manufacturer. RNA quality and integrity were analysed (Agilent® 2100 Bio-analyzer, Agilent) and RNA was treated with DNase 1 to remove gDNA contamination. For sequencing purposes, mRNA was synthesized from 2 µg of total RNA by an already-described method^46^. A TruSeq^TM^ RNA library prep kit (Illumina) was used to generate RNA libraries. Single-stranded cDNA was synthesized using hexamer priming of mRNA to generate double stranded cDNA libraries, which were then quantified using a KAPA library quantification kit (Illumina) and sequenced through a HiSeq-2500 sequencer (Illumina).

### Cufflinks and Cuffdiff analysis for identifying Differentially Expressed Genes (DEGs)

Raw sequence reads (low quality reads with adaptors included) were further processed to identify high quality reads. The threshold level for high quality reads was Q20 > 40%; and the reads less than the threshold level or having more than 10% ambiguous bases were removed. The reads were further processed for high quality using a customized analysis program developed by Theragen ETEX (Korea). The high-quality reads obtained were compared to the reference *Arabidopsis thaliana* genome using Ensemble.

The reads were then aligned with the reference genome through TopHat^55^, using default values. After aligning the reads, we inferred the abundance of the transcripts using Cufflinks package v2.2.1^56^. Cufflinks assembles the fragments and calculates the transcript abundance based on the number of reads. The gene and transcript expression levels were calculated for control and treated conditions and were tested for significant differences using Cuffdiff v.2.2.1^56^ to identify differentially expressed genes (DEGs). The genes having significant differential expression (Q < 0.05) were selected. A Heatmap showing the hierarchical clustering of differentially expressed genes was generated using *R* (https://www.r-project.org/).

### Identification, annotation, and Gene Ontology analysis of transcription factors

The selected DEGs were analysed for annotation and GO terms using the NCBI (www.ncbi.nlm.nih.gov) and Gene Ontology Consortium databases (http://geneontology.org/). All the DEGs were then mapped against the *Arabidopsis* “Ath_AGI_LOCUS_TAIR10_Aug2012.m02” database in MapMan^57^ to identify transcription factors (TFs). MapMan identifies TFs based on its comparison with the reference genome. The TFs thus obtained were validated manually using the annotated list of all the DEGs and were analysed for any duplicated values using conditional formatting in Microsoft EXCEL. A Heatmap representing the hierarchical clustering of expression differences between treated and control samples using FPKM values was generated using *R* (version 3.3.1). Dispersion in the data was analysed using a Multi-Dimensional Scatter (MDS) plot using *R* (version 3.3.1). Further, the identified TFs were analysed for functional classification through the PANTHER classification system using *Arabidopsis thaliana* as reference genome.

### Classification of transcription factors into families and pathways using MapMan analysis

RNA-seq data, usually comprising of several thousand genes and transcripts, is challenging to analyse efficiently. To study the involvement of NO-responsive transcription factors in different pathways and cellular processes, we analysed all TFs with their expression values using MapMan version 3.6.0RC1. MapMan is an omics data analysis software that allows visualization of omics data using a hierarchical BIN-based ontology system^46^.

To study in detail the involvement of NO-responsive TFs, we mapped all the TFs (Q < 0.05) against the already-mapped *Arabidopsis* database (TAIR 10 Aug 2012) in MapMan. The analysed pathways and cellular processes were then collectively mapped using their BIN and sub-BIN numbers on custom-made images to present in concise and succinct form.

### Oxidative and nitrosative stress assay

To examine the role of TFs in oxidative and nitrosative stress tolerance, we selected three different TFs one each from different categories including those involved in defence (RAP2.6; At1g43160 an ERF TF as majority of defence related TFs were related to ERF family), growth and development (DDF1; At1g12610) and secondary metabolite production (MYB48, At3g46130 involved in flavonol biosynthesis) based on their annotation and fold change (all the selected candidates having more than 25 fold change) for studying downstream functions through functional genomics. The mutant lines *ddf1*, *rap2*.*6*, and *atmyb48* were genotyped at the rosette leaf stage in order to identify homozygous lines. *Arabidopsis* WT and all other lines used in the study were of the Col-0 genetic background. Plants were tested for different growth parameters under nitrosative and oxidative stress conditions. All the seeds were surface sterilized and germinated on half-strength MS medium in triplicate, containing 2 mM H_2_O_2_ (for oxidative stress) or 1 mM S-nitrocysteine (CySNO) (for nitrosative stress), as described previously^58^. Data on cotyledon development frequency (CDF), shoot length, and root length were recorded 2 weeks after treatment started. The term CDF was used to mention the number of developed, green seedlings as in case of nitrosative stress seeds of mutants that lack NO scavenging mechanism (e.g *atgsnor 1-3*) may germinate but cannot survive.

### Pathogenicity assessment

The virulent *Pseudomonas syringae* pv. *tomato* strain DC3000 and *avirulent Pst* DC3000 expressing the *avrB* effector were grown and maintained, as described previously^26^. Briefly, the bacterial strains were grown on LB (Luria-Bertani)-agar media with appropriate antibiotics for selection (rifampicin for virulent *Pst* DC3000 and kanamycin and rifampicin for *Pst* DC3000 (*avr*B). Following this, 5 mL of liquid culture was made using a single colony in LB broth and incubated at 28 °C overnight. Both bacterial strains were then harvested by centrifugation at 13,000 rpm for 1 min in 10 mM MgCl_2_, and syringe-infiltrated into the abaxial side of leaves at rosette leaf stage at a concentration of 5 × 10^5^ CFU mL^−1^. Control plants were only infiltrated with 10 mM MgCl_2_. Leaf samples (1 cm each) from plants inoculated either with *Pst* DC3000 or *Pst* DC3000 (*avrB*) were collected at two and four days post inoculation (DPI) and crushed in 1 mL of sterile 10 mM MgCl_2_. The homogenate was diluted by a factor of 10 and spread on LB-agar plates containing appropriate antibiotics. Plates were incubated at 28 °C for 48 h and colonies were counted.

### Validation of RNA-seq data through qRT-PCR analysis

To validate the expression level of TF genes that showed differential expression in response to CySNO in RNA-seq mediated transcriptome analysis, we selected representative genes involved in key processes like growth and development, signalling, defence, protein degradation etc. for quantitative real time PCR (qRT-PCR) analysis. A detailed list of the genes and primers is given in supplementary Table S5. For qRT-PCR analysis, RNA was extracted using Trizol (Ambion, Life Technologies USA). The RNA concentration was measured using nanoQ (OPTIZEN Korea), and 2 µg of total RNA with good integrity and purity was used to synthesize cDNA using a DiaStar^TM^ RT Kit (SolGent, Korea). To measure transcript accumulation, a two-step qRT-PCR reaction was performed using the Illumina (USA) Eco^TM^ real time PCR system using a 2× Quantispeed SYBR Kit (PhileKorea) according to the manufacturer’s recommended protocol.

## Electronic supplementary material

Dataset 1

Dataset 2

Dataset 3
