# Supplementary material for: Transcriptome profile of NO-induced Arabidopsis transcription factor genes suggests their putative regulatory role in multiple biological processes
Source: Sci Rep. 2018 Jan 15;8:771. doi: 10.1038/s41598-017-18850-5 (PMC5768701; doi:10.1038/s41598-017-18850-5)
Supplement: Supplementary file 1 — Dataset 1 [file 41598_2017_18850_MOESM1_ESM.doc]

**Transcriptome profile of NO-induced Arabidopsis transcription factor genes suggests their putative regulatory role in multiple biological processes**

**Qari Muhammad Imran^1^, Adil Hussain^2^, Sang-Uk Lee^1^, Bong-Gyu Mun^1^, Noreen Falak^1^, Gary J. Loake^3*^ and Byung-Wook Yun^1^***

^1^Laboratory of plant functional Genomics, School of Applied BioSciences, Kyungpook National University, Daegu, Republic of Korea.

^2^Department of Agriculture, Abdul Wali Khan University, Mardan, Pakistan

^3^Institute of Molecular Plant Sciences, University of Edinburgh, King’s Buildings, Edinburgh, UK

***Corresponding Authors:** [**bwyun@knu.ac.kr**](mailto:bwyun@knu.ac.kr)**,** [**gloake@ed.ac.uk**](mailto:gloake@ed.ac.uk)


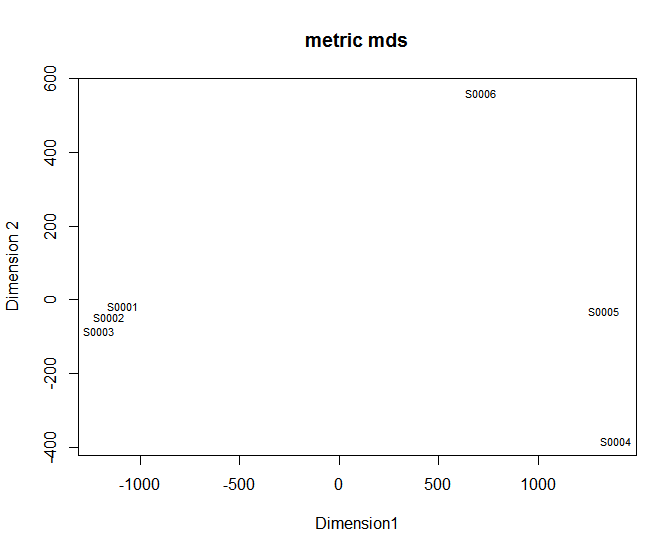


**Supplementary Figure. S1**. Multi-dimensional scaling (MDS) plot showing dispersion among the FPKM values of NO-responsive TFs. The labels S001, S002 and S003 represents control treatments while S004, S005 and S006 represents CySNO treatments.


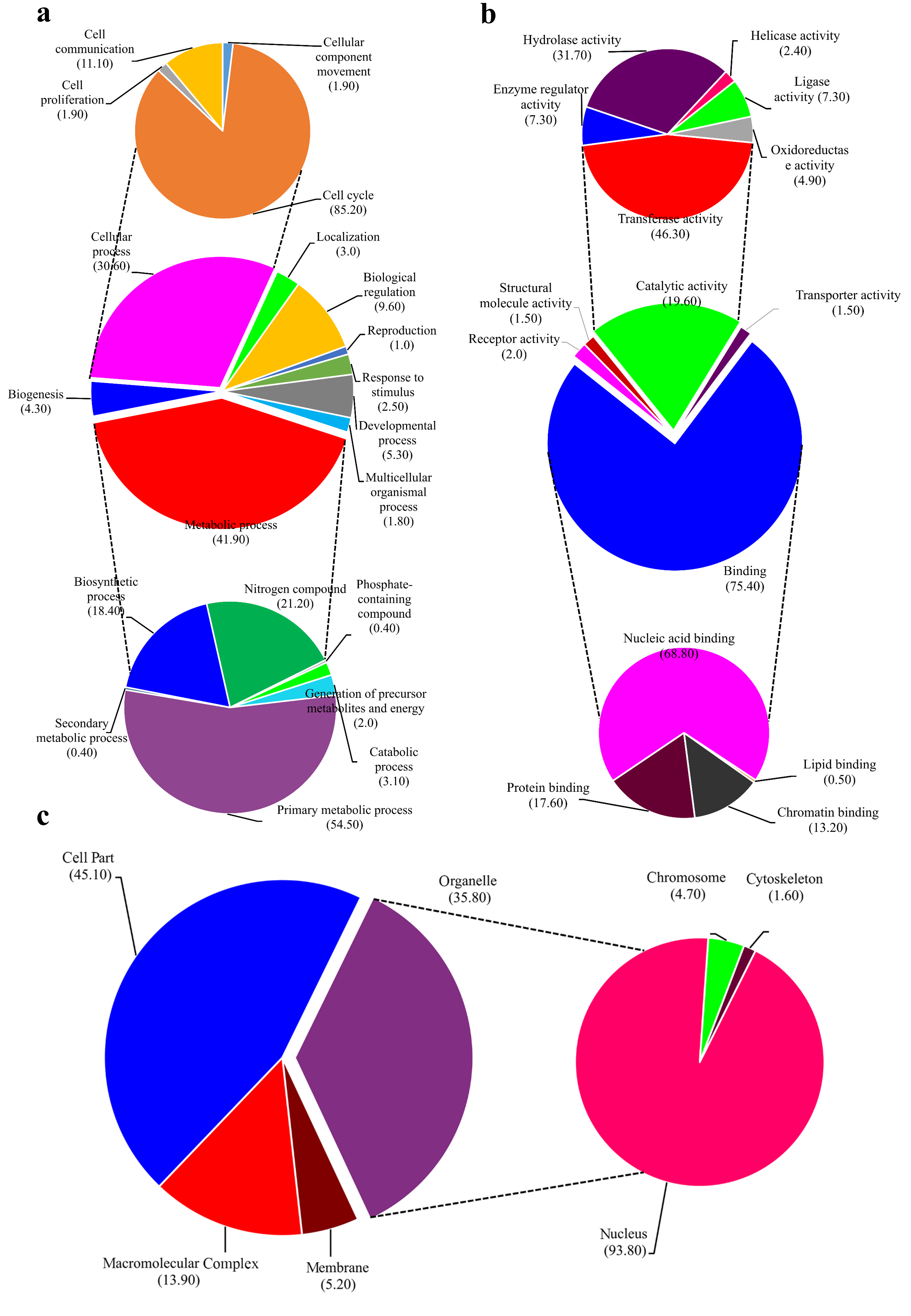


**Supplementary Figure. S2**. Gene ontology classification of CySNO responsive TFs in *Arabidopsis* leaf. All the TFs were analysed for GO-terms using the PANTHER classification system (<http://pantherdb.org/>). Gene ontologies for (a) Biological Processes (b) Molecular Functions and (c) Cellular Components were determined using *Arabidopsis thaliana* as a reference genome. Out of 673 of total CySNO responsive TFs, 664 were successfully mapped to the reference genome..


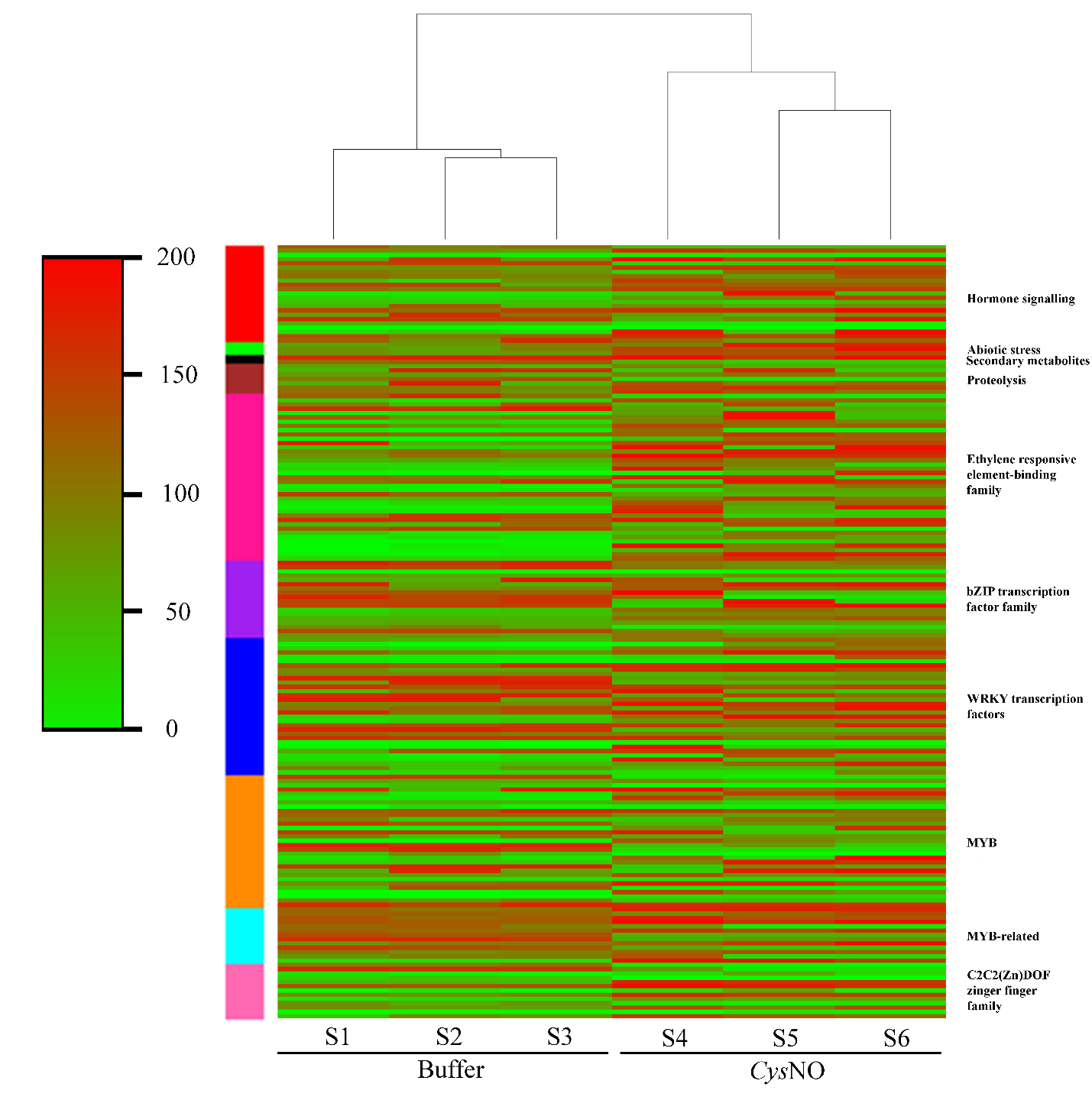


**Supplementary Figure. S3**. Heat map representing FPKM values of TFs involved only in defense.


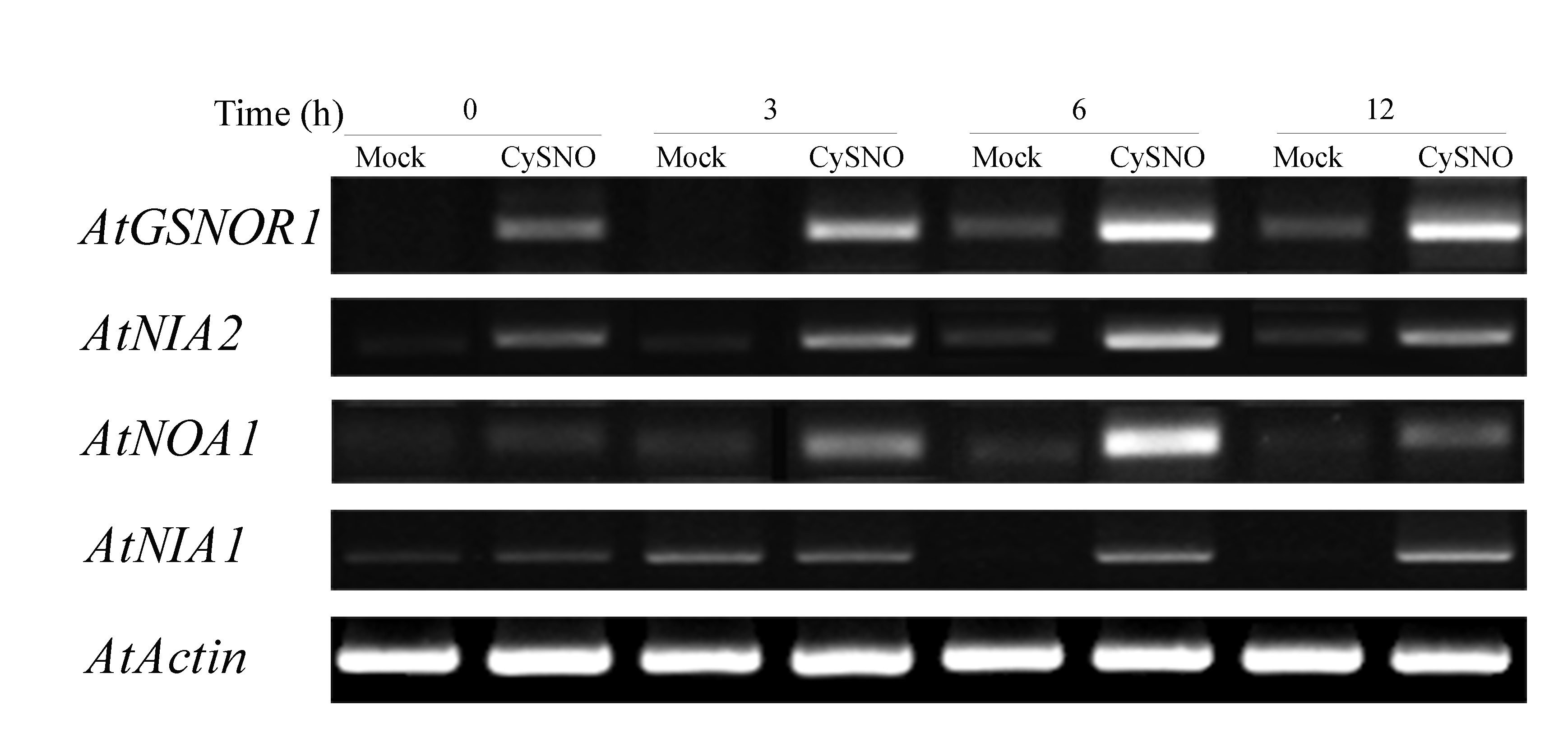


**Supplementary Figure. S4**. RT-PCR for NO-signaling related genes after 0, 3, 6 and 12h of 1mM CySNO infiltration. Mock treatments were used as comparative control. The gel pictures were cropped and combined for better presentation. The original gel images are presented in supplementary Fig. S6.


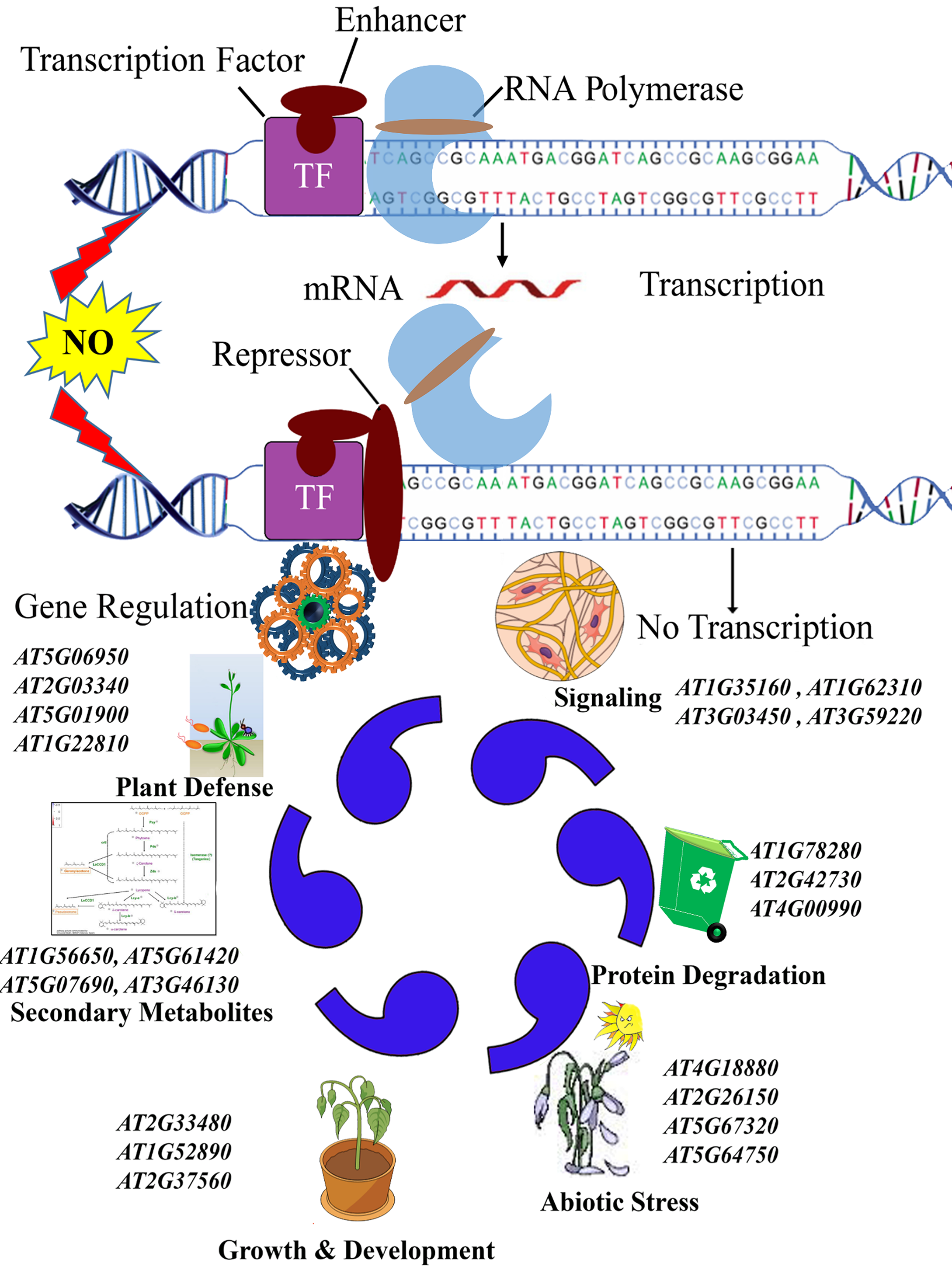


Supplementary Figure. S5. Schematic representation and working model of NO-induced transcription factors (TFs).

The upper part of the figure represents the binding capacity of NO-induced TFs that may either bind to enhancer proteins, enabling the recruitment of RNA-polymerase to start transcription that will lead to protein synthesis, or a repressor protein that will ultimately block the RNA-polymerase attachment and hence, no transcription will occur. The bottom part represents NO-induced TFs that were validated through qRT-PCR and are involved in different biological processes like signalling, protein degradation, abiotic stress tolerance, growth and development, secondary metabolite production, and plant defence. The illustrations used in this figure were made through a combination of Microsoft PowerPoint (2013) and ConceptDraw office (10.2.0.2) programs.

Supplementary Table S2. List of the top 10 up-regulated TF genes that showed differential expression in response to 1mM CySNO in an RNAs-seq based transcriptome in *Arabidopsis thaliana*.

| **Acc. No** | **Name** | **FPKM(1)** | **FPKM(2)** | **Log2( Fold change)** | **P-value** | **Annotation** ^1^ |
| --- | --- | --- | --- | --- | --- | --- |
| **AT1G71520** | **_** | 0.02000 | 38.18000 | 10.83590 | 0.00002 | Encodes a member of DREB subfamily. The protein contains one AP2 domain. |
| **AT2G22760** | **_** | 0.04000 | 44.05000 | 10.18870 | 0.00000 | Basic helix-loop-helix (bHLH) DNA-binding superfamily protein; involved in regulation of transcription |
| **AT1G22810** | **_** | 0.15000 | 153.56000 | 9.96553 | 0.00000 | Encodes a member of DREB subfamily A-5 having one AP2 domain |
| **AT1G43160** | RAP2.6 | 0.40000 | 332.81000 | 9.70626 | 0.00000 | Encodes a member of the ERF (ethylene response factor) subfamily B-4 |
| **AT5G64750** | ABR1 | 0.65000 | 535.74000 | 9.68623 | 0.00000 | Member of ERF subfamily. Involved in ABA signaling pathway |
| **AT3G53600** | **_** | 0.08000 | 36.62000 | 8.79631 | 0.00514 | C2H2-type zinc finger family protein; involved in response to chitin, regulation of transcription |
| **AT4G29930** | **_** | 0.08000 | 28.25000 | 8.39629 | 0.00000 | Basic helix-loop-helix (bHLH) DNA-binding superfamily protein; involved in regulation of transcription |
| **AT4G28140** | **_** | 0.41000 | 120.81000 | 8.20867 | 0.00000 | Encodes a member of DREB subfamily A-6 having one AP2 domain; involved in regulation of transcription |
| **AT2G40340** | DREB2C | 0.04000 | 7.83000 | 7.70854 | 0.00000 | Encodes a member of DREB subfamily A-2 having one AP2 domain |
| **AT4G05100** | AtMYB74 | 0.47000 | 95.02000 | 7.66846 | 0.00000 | Member of the R2R3 factor gene family. |

^1^The annotations are based on information from TAIR (https://www.arabidopsis.org/index.jsp)

Supplementary Table S3. List of the top 10 down-regulated TF genes in the RNA-seq based transcriptome of *Arabidopsis* leaf infiltrated with 1mM CySNO.

| **Acc. No** | **Name** | **FPKM(1)** | **FPKM(2)** | **Log2( Fold change)** | **P-value** | **Annotation** |
| --- | --- | --- | --- | --- | --- | --- |
| **AT3G52910** | AtGRF4 | 0.41000 | 0.01000 | -4.95192 | 0.00668 | Growth regulating factor encoding transcription activator. Involved in leaf development |
| **AT4G32280** | IAA29 | 4.87000 | 0.17000 | -4.88034 | 0.00000 | Auxin inducible protein |
| **AT5G03150** | JKD | 1.29000 | 0.04000 | -4.84552 | 0.00001 | a nuclear-localized zinc finger domain containing transcription factor |
| **AT3G46130** | ATMYB48 | 50.75000 | 1.81000 | -4.80639 | 0.00000 | Encodes a putative transcription factor that functions to regulate flavonol biosynthesis primarily in cotyledons |
| **AT1G73830** | BEE3 | 5.04000 | 0.22000 | -4.50709 | 0.00000 | BR enhanced expression 3 (BEE3); involved in DNA binding |
| **AT3G55734** | MIR393B | 0.67000 | 0.03000 | -4.48027 | 0.00001 | Encodes a microRNA that targets several TIR1/AFB family members and one bHLH family member |
| **AT1G64625** | - | 0.49000 | 0.03000 | -4.24715 | 0.00523 | Serine/threonine=protein kinase WNK (with no Lysine) |
| **AT4G30410** | AT4G30410 | 8.04000 | 0.43000 | -4.22033 | 0.00129 | Sequence-specific DNA binding transcription factors |
| **AT1G11850** | - | 4.33000 | 0.24000 | -4.18487 | 0.00725 | Unknown protein |
| **AT4G36540** | BEE2 | 59.26000 | 3.30000 | -4.16750 | 0.00000 | Encodes the brassinosteroid signaling component BEE2. Positively regulates the shade avoidance syndrome in Arabidopsis seedlings. |

Supplementary Table. S5. List of TF genes that were validated through qRT-PCR and their primer sequences.

| **Gene Accession** | **Primer Forward (5' - 3')** | **Primer Reverse (5'-3')** |
| --- | --- | --- |
| AT4G18880 | AATGGGAATTTGCGAATGAT | TCTCACTCGTTCTGAATCCG |
| AT2G26150 | ATGGAGGAAGAAACGGTGAC | CTGTTTCGTTAAGCCCTTCC |
| AT5G67320 | GATTGGCGCAACAATGTATC | CCTGATGCCCAGTAAAGGTT |
| AT1G01010 | TCTGATTGGGTTATCCACGA | GGGAGTGGGATCTATTGCAT |
| AT1G52890 | TTCTCGTAGAAACGGAAGCA | ACGAAGTACCGTTGTTGCTG |
| AT3G04070 | CGATGAGACTGGATGATTGG | TTCCTCATGCTCTTGATTGC |
| AT2G33480 | GCTAAATACCCGAACGGAAA | CGATCTGCTTATCGAGACCA |
| AT2G37560 | CATCAATGGCTACCTCCCTT | ATATCATCCATGGAGCGTGA |
| AT1G62310 | AGCTAGGTGAAGCGGTGTTT | TGACACAAAGTCAACTGCGA |
| AT1G78280 | GCACACTACTACCTGGCGAA | CATATCCAAGCACACGAACC |
| AT2G42730 | TATTGGGAATGACAAGGCAA | GACCCATGAAGATGAGGGTT |
| AT4G00990 | GAGAGGGAAGCGAATCAGAC | CGATTCTTCCTTTGCTCACA |
| AT5G54630 | TTGGTTAACGGTGGAGATGA | GCCAAAGAATCAAACCCACT |
| AT1G56650 | TGGCACCAAGTTCCTGTAAG | AAGCCTATGAAGGCGAAGAA |
| AT5G61420 | ATCATGCTTCATGCTTCTCG | TTGTGAGTCACGGGATCAAT |
| AT5G07690 | TGTACCGGAGATAACCAGCA | TAGTTCCCATGGAAGAAGCC |
| AT3G03450 | CCGAATACGATCTCAGAGCA | CCTCTTGCTGCTAGCTTCCT |
| AT1G35160 | GAGTTCGTGTACCTCGCAAA | AAGCGACGGAGAGGAGATTA |
| AT2G32250 | TCGATCAGTGGGATTTGGTA | GGTCTTTGGACATGAACGTG |
| AT3G59220 | GAAGGAAGGTGAAGGAGCTG | TCTGTGAGGATGATCTGGGA |
| AT1G12610 | TAAGGCGGAGGAACGGTGAC | GCGCTGCCATATCTGCTGTG |
| AT3G46130 | CCATGGACAGAACAGGAAGA | ACCCATCTTAGCCTGCAACT |
| AT1G22810 | TCTCCGAGATCTATCCAGCA | TGAAATATTCAACGGCTCCA |
| AT5G64750 | AGAGTTTGGCTCGGTACGTT | AGTTTGGCTTTGTTGCCTCT |
| AT5G06950 | TGGGCATAAATAACCTGCAA | TCCCTGATGAACTTGAACCA |
| AT2G03340 | GGGTCAGAGAGAGACTTCGG | CGCCAGTTGTATCCATCATC |
| AT5G01900 | CCAACCAGCTGCTCATCATG | GGCCAAATCCTCCCTTTCC |
| AT3G18780 | GCTGGACGTGACCTTACTGA | CCATCTCCTGCTCGTAGTCA |
| AT1G43160 | GCGGAGATTCGAGACCCACA | CGAAGTGCTGCCGCATCATA |
| *AtNIA1* | AGTACGGTAAATTCTGGTGCTGGTG | CCCTATCTCTCCTCTATGAGGCTTG |
| *AtNIA2* | GACGCCGAACTCGCCGACGAAG | TGTCTCTCCACCATCTACCGTGACCTC |
| *AtNOA1* | CGAAAGGGATCCTGTTGCAG | ACCCGGTGTGTCATACAACT |
| *AtGSNOR* | CGTCGCCAAAATTGATCCTACTGC | TCACTTCCTGAATTGGCTTGTCGT |
| *ABA2* | ACGGTTGATGATGTAGCGAACGCTGTT | CATCTGAAGACTTTAAAGGAGTGGTTAG |
| *ABI2* | GTTCTTGTTCTGGCGACGGAGC | CCATTAGTGACTCGACCATCAAG |
| *NCED3* | TCCTCTGTTTCGTTCACGACG | CGTACGGAACCCTTGACGGA |
| *NOX1* | GGGCACATTTAGCGTGTGAT | CGTGGCTAGTGTGAATGCAA |
| *PR1* | GTGCAATGGAGTTTGTGGTC | TCACATAATTCCCACGAGGA |
| *PR2* | CAGATTCCGGTACATCAACG | AGTGGTGGTGTCAGTGGCTA |
| *Actin* | AGGTTCTGTTCCAGCCATC | TTAGAAGCATTTCCTGTGAAC |


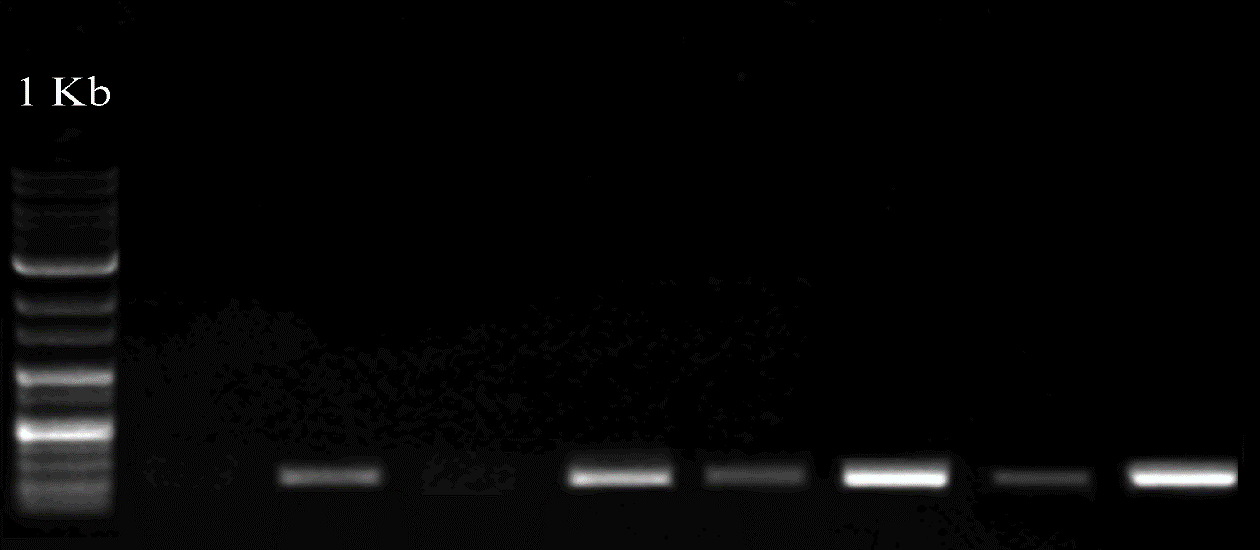


a


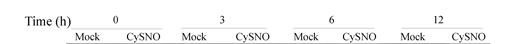


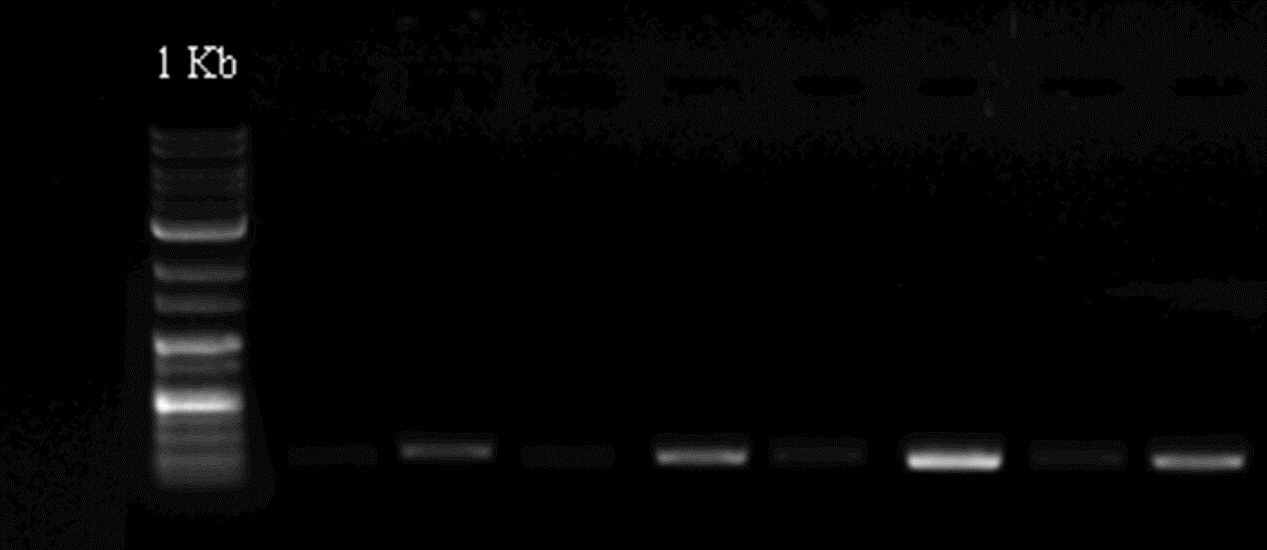


b


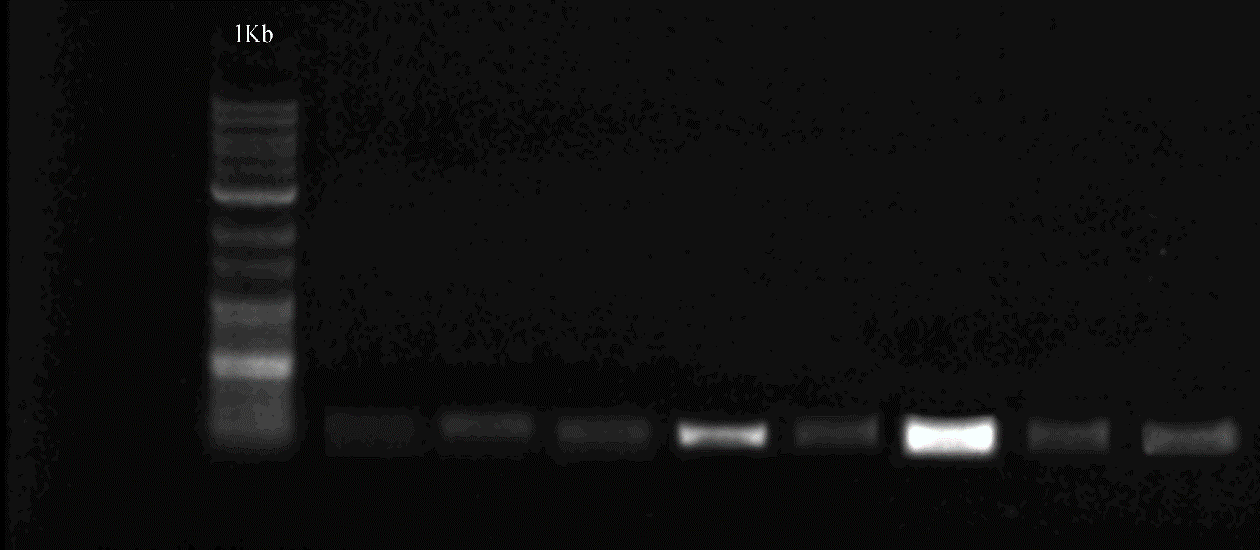


c


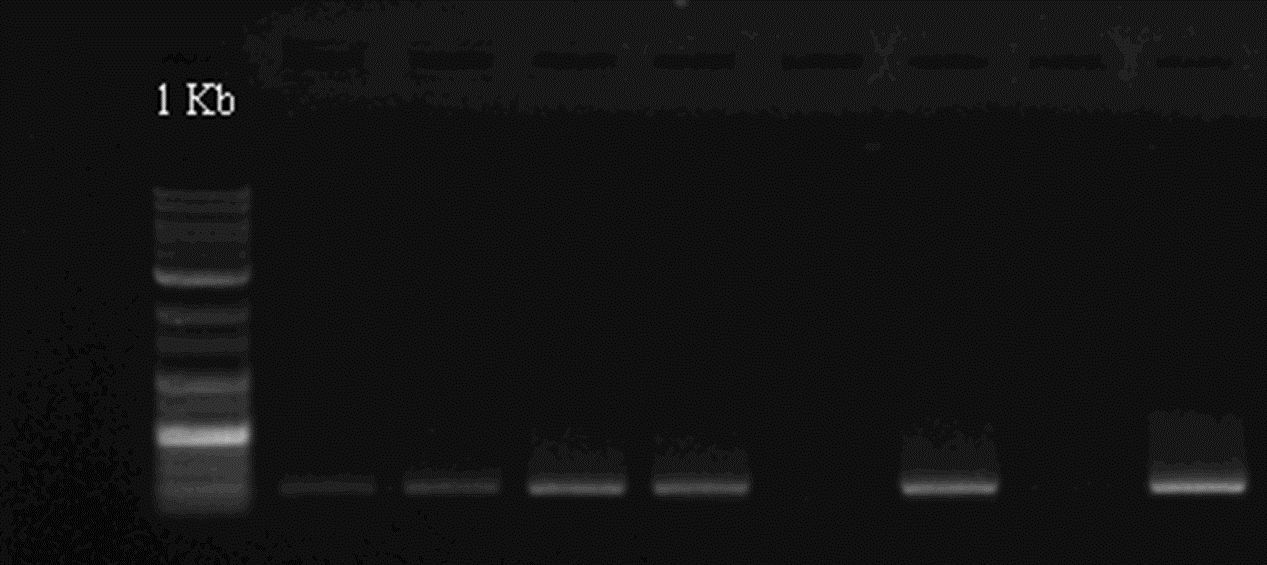


d


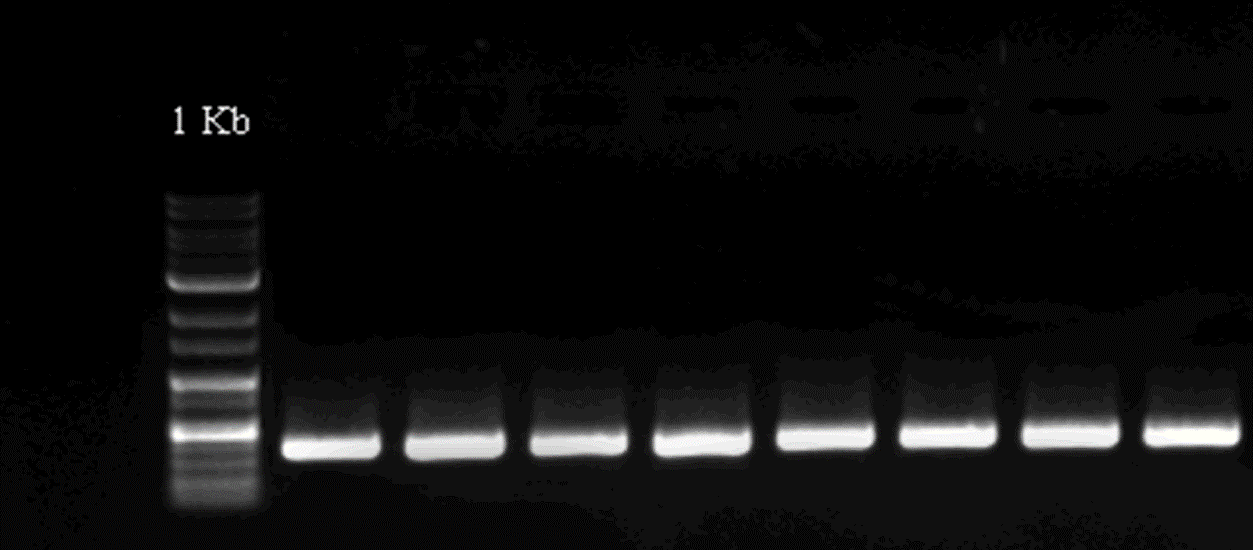


e

Supplementary Figure S6. Orignal gel images of (a) *AtGSNOR1* (b) *AtNIA2* (c) *AtNOA1* (d) *AtNIA1* (e) *AtActin* taken with UVITEC (CAMBRIDGE UK) software. Extra spaces were cropped to reduce image size.
